# eDNA metabarcoding for biodiversity assessment, generalist predators as sampling assistants

**DOI:** 10.1038/s41598-021-85488-9

**Published:** 2021-03-25

**Authors:** Louise Nørgaard, Carsten Riis Olesen, Kristian Trøjelsgaard, Cino Pertoldi, Jeppe Lund Nielsen, Pierre Taberlet, Aritz Ruiz-González, Marta De Barba, Laura Iacolina

**Affiliations:** 1 Department of Chemistry and Bioscience, Aalborg University, Fredrik Bajers Vej 7H, 9220 Aalborg, Denmark; 2 School of Biological Sciences, Monash University, 18 Innovation Walk, Melbourne, 3800 Australia; 3 Danmarks Jægerforbund, Molsvej 34, 8410 Rønde, Denmark; 4 Aalborg Zoo, Mølleparkvej 63, 9000 Aalborg, Denmark; 5 CNRS, Laboratoire D’Ecologie Alpine (LECA), Univ. Grenoble Alpes, 38000 Grenoble, France; 6 UiT – The Arctic University of Norway, Tromsø Museum, Hansine Hansens veg 18, 9019 Tromsö, Norway; 7 Department of Zoology and Animal Cell Biology, University of the Basque Country UPV/EHU, C/Paseo de la Universidad 7, 01006 Vitoria-Gasteiz, Spain; 8 Lascaray Research Center, University of the Basque Country (UPV/EHU), Avda. Miguel de Unamuno, 3, 01006 Vitoria-Gasteiz, Spain; 9 Department of Biodiversity, University of Primorska, Glagoljaška 8, 6000 Koper, Slovenia

**Keywords:** Biological techniques, Ecology, Molecular biology

## Abstract

With an accelerating negative impact of anthropogenic actions on natural ecosystems, non-invasive biodiversity assessments are becoming increasingly crucial. As a consequence, the interest in the application of environmental DNA (eDNA) survey techniques has increased. The use of eDNA extracted from faeces from generalist predators, have recently been described as “biodiversity capsules” and suggested as a complementary tool for improving current biodiversity assessments. In this study, using faecal samples from two generalist omnivore species, the Eurasian badger and the red fox, we evaluated the applicability of eDNA metabarcoding in determining dietary composition, compared to macroscopic diet identification techniques. Subsequently, we used the dietary information obtained to assess its contribution to biodiversity assessments. Compared to classic macroscopic techniques, we found that eDNA metabarcoding detected more taxa, at higher taxonomic resolution, and proved to be an important technique to verify the species identification of the predator from field collected faeces. Furthermore, we showed how dietary analyses complemented field observations in describing biodiversity by identifying consumed flora and fauna that went unnoticed during field observations. While diet analysis approaches could not substitute field observations entirely, we suggest that their integration with other methods might overcome intrinsic limitations of single techniques in future biodiversity surveys.

## Introduction

Anthropogenic influences on ecosystems are currently causing devastating changes and we are now observing increased rates of species extinction, loss of biodiversity and, as a consequence, loss of ecosystem functioning^1^. Thus, rapid, habitat-specific and non-invasive biodiversity assessments are urgently needed^2^. Biodiversity has been assessed, at varying geographic scales, by a variety of methods such as flight trapping, pitfall traps^3^, acoustic surveys^4^, camera traps^5^ and field observations^6^. While these methods have been designed for application to specific environments, study systems, and species, they have several drawbacks and limitations. For example, approaches based on visual observation and acoustic detection are highly dependent on the identification skills of the operator^7^. Additional challenges are constituted by biases in overall species occurrence, seasonality, behaviour and changes in community composition due to alternation in species distribution (e.g. sink or marginal areas) or to environmental fluctuations (e.g. temporal ponds, succession stages)^8^. Physical survey methods (e.g. trapping) are invasive, and therefore often not applicable when dealing with species of conservation concern. Furthermore, conventional methods (as described above) are constrained by a species behaviour and body size, and are highly time consuming^9^. As a consequence, biodiversity survey techniques that are less dependent on direct behavioural observations, and are less invasive have gained increasing interest^10,11^.

In particular, amplicon sequencing of DNA from various sources of environmental samples (eDNA), using a metabarcoding approach, allows for the taxonomical characterisation of organismal matter contained in complex mixtures such as soil, water, air, stomach content and animal faeces^12,13^. A major benefit of eDNA metabarcoding for biodiversity surveys, compared to earlier mentioned methods, is that it enables efficient and non-invasive species detection with relatively small effort (and continuously decreasing price), even for large scale surveys^12,14^. eDNA metabarcoding is now widely applied to various terrestrial and aquatic samples, both ancient and modern, showing great potential for use in biodiversity monitoring and ecological studies^10,11,13^. For example, specific ecological applications include verifying species presence and potential range expansion^15,16^, uncovering drivers of community composition^17,18^ and estimating species richness and diversity^10,19,20^. In addition, the method has proven highly useful for diet analysis^21,22^ and derived applications such as unravelling food webs^23–25^, predator–prey interactions^18,26^, herbivore ecosystem function^14,27^ and niche breadth^28,29^.

Among the possible eDNA sources for diet analysis, faeces from generalist predators have recently been described as “biodiversity capsules” and a complementary tool for improving current biodiversity assessments^30,31^. This concept stems from the fact that generalist predators and omnivore species forage on a large variety of food resources in their habitat. Therefore, compositional studies of their faeces may reveal a broad spectrum of the species occurring within their home range. Only few studies so far have incorporated DNA based diet composition information from generalist, opportunistic and omnivore predator species in biodiversity assessments^30,32,33^. Studies using both faecal and stomach content have shown the applicability of eDNA metabarcoding to detect consumed flora and fauna biodiversity, with particular advantage in environments that are difficult to sample, since this technique can identify more species than traditional methods^34,35^. However, previous work also identified some important pitfalls of faecal dietary analysis in ecological surveys. Specifically, geographic distance and time lag between consumption of the prey and deposition of the faeces can hamper assumptions on spatial and temporal patterns of species presence^32,35^, but also the time from faecal dropping to collection, as well as the substrate, can influence detection accuracy of eDNA methods^36^. An additional important aspect is that, while predator diet is generally assumed to reflect abundance and diversity of prey species in the predator foraging area, the diet is also influenced by predator behaviour and preferences in its ecosystem, which will, to varying extent, bias detection of the species present in the study area^31^. Validation studies, including parallel comparisons of multiple survey methods, and evaluation of the detection bias in areas with existing ecosystem monitoring data are therefore important in order to assess the usefulness of faecal compositional analysis as a complementary biodiversity monitoring tool.

With this in mind, we empirically evaluate if, and to what extent, eDNA metabarcoding analysis of faecal samples from generalist predators collected non-invasively can be used as a tool for characterising and monitoring the biodiversity at a particular study site. To do this, we focus on two predatory generalist omnivore species, the Eurasian badger (*Meles meles*) and the red fox (*Vulpes vulpes*) in a protected area in Denmark, for which a comprehensive species inventory list is available, providing an overview on the areas long-term biodiversity. Specifically, we: i) use eDNA metabarcoding on non-invasively collected faecal samples to assess the diet of the two omnivorous predatory species by applying universal markers covering the plant, vertebrate and invertebrate components of the diet, ii) assess the effect of environmental exposure (faecal age since deposition) on eDNA metabarcoding detection efficiency, iii) compare eDNA metabarcoding results to macroscopic diet assessment, iv) compare the list of prey species detected in the diet by eDNA and macroscopic identification techniques to the comprehensive species list achieved by traditional field observations.

## Methods

### Study area and sample collection

**Figure 1:**
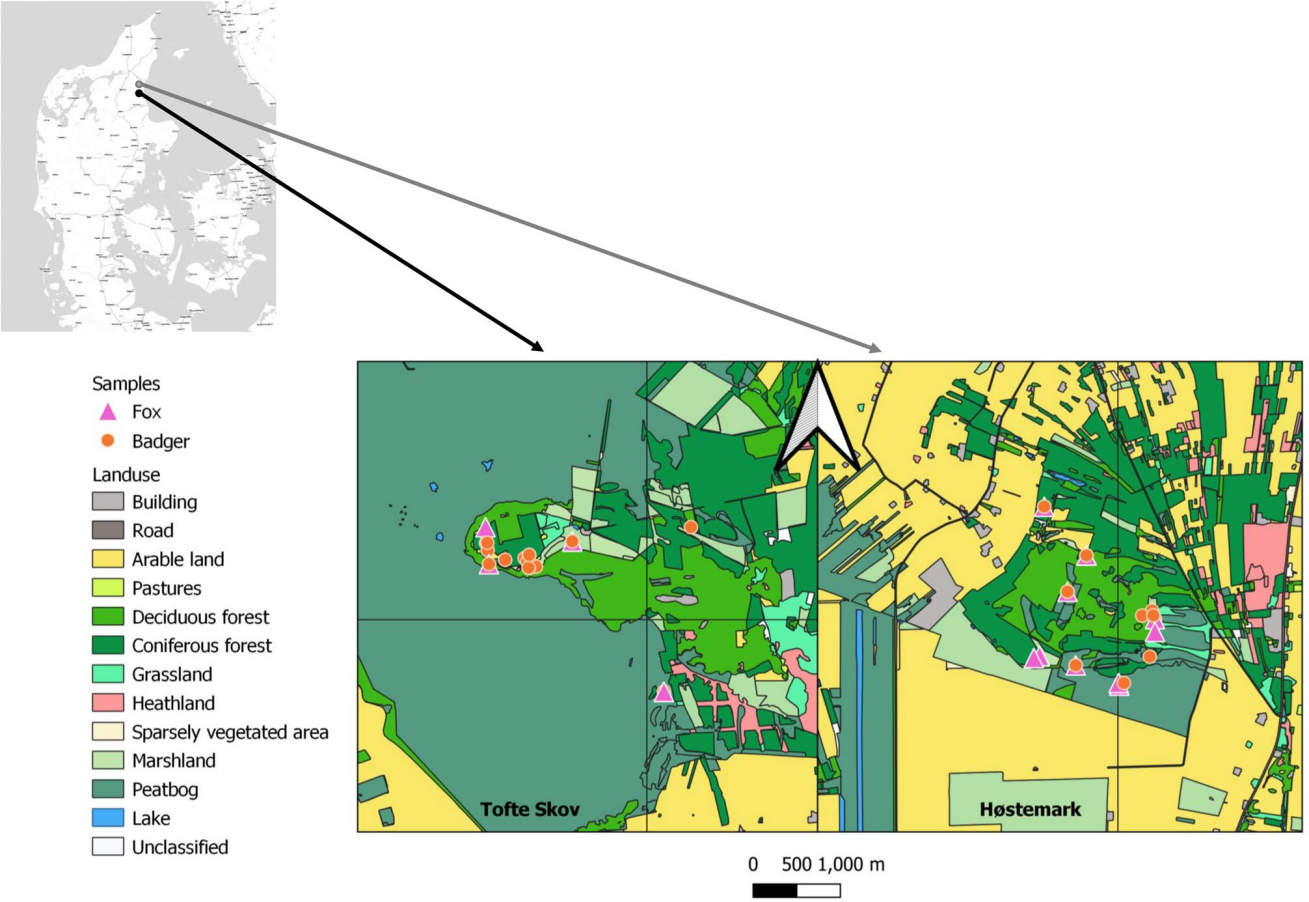
Location of the sampling area, Lille Vildmose in Denmark. Høstemark skov (grey dot in top left corner) and Tofte Skov (black dot in top left corner). Maps were created using qGIS ver. 3.16 (https://www.qgis.ord/en/site).

Faecal samples from foxes and badgers were collected from April to December 2015 in Lille Vildmose, Denmark (56°53′15″N 10°13′14″E; Fig. 1). Permission needed for survey were obtained from concerned institutions including Aage V. Jensen’s foundation. The area covers 8000 ha and is separated into two fully protected and fenced areas (Høstemark 568 ha; and Tofte Skov 4000 ha), and two areas open to the public (Portlandsmosen and Paraplymosen). In this study, we surveyed the two fully protected areas where human impact is minimal. Sampling within these fenced areas ensured that the faecal samples included in the analysis were unlikely to contain prey species from outside of the intended sampling area (e.g. movement and foraging was limited to within the fenced area), and prey species retained from faecal content were therefore directly comparable with field observations from the study area. Recent research has documented that the permanently fenced (since 1906) and grazed forests of Tofte Skov and Høstemark (since 1933) contain an unusually high biodiversity compared to other areas in Denmark^37^, and is therefore of particular interest.

Surveys were performed weekly (with few exceptions) from April to December 2015 in both areas and were primarily focused on locations where installation of camera-traps had verified the presence of the focal predators. Surveys were performed by trained personnel with good skills in distinguishing wildlife faecal matters. Although other species with morphologically similar faecal structures are potentially present in the area (e.g. raccoon dog, *Nyctereutes procyonoides*, or various mustelid species like American mink, *Mustela neovison* or marten species such as *Martes martes*), badger faeces are characterized by their deposition in latrines^38^ whereas fox faeces are usually identified by their characteristic structure and deposition at marking locations. While the deposition of faecal material in latrines can lead to sample non-independence for badger, this bias would not affect conclusions of biodiversity estimates for the study area. The age (days since deposition) of collected faecal samples was estimated based on visual inspection and time since the previous survey and was used to pool the samples into two age categories (< 7 and > 7 days). The latter information was used to assess if DNA degradation, due to longer sample exposure to environmental factors, has major effects on DNA metabarcoding results and can therefore compromise the use of predator faeces for biodiversity assessments.

Upon collection, all samples were placed in a cooler with ice elements and subsequently transferred to − 20 °C until analysis. Subsamples for eDNA metabarcoding were taken on site and transferred to 1.5 mL Eppendorf tubes using clean, single use, spatulas. The outer layer of each faeces was gently removed and samples were taken from the inner part of the faeces to reduce environmental contamination, as recommended elsewhere^36,39^. The remaining faecal sample was transferred to plastic bags for macroscopic diet analysis.

### eDNA metabarcoding

DNA extraction from collected faecal samples was performed following a protocol described elsewhere^40^ with minor modifications (see Supplementary Note 1). Diet analysis was performed by co-amplifying three universal markers, targeting short (< 100 bp) and variable DNA fragments of the plant (on the P6 loop of the chloroplast trnL (UAA) intron), vertebrate (on the V5 loop of the mitochondrial 12S gene) and invertebrate (on the mitochondrial 16S gene) components of the diet (see Supplementary Note 1 for details and marker references). For simplicity, universal markers will throughout the paper be referred to as the target diet categories *i.e.* plants, invertebrates and vertebrates. Blocking oligonucleotides were used to minimize amplification of badger, fox and human sequences with the vertebrate marker, and of mammalian and human sequences with the invertebrate marker (Supplementary Note 1 and Table 1). For taxonomic classification, a sequence reference database was built for each eDNA metabarcoding marker by extracting the relevant DNA region for plants, vertebrates, and invertebrates (*Arthropoda* and *Mollusca*) from EMBL nucleotide library (release 128) using the ecoPCR program^41^. Sequence reads were filtered and taxonomically identified following the procedure described in^39^ using the OBITools^42^ and R v. 3.3.3^43^ with modifications described in Supplementary Note 1.

**Table 1:** Overview of number of detections at the order and species level, using eDNA metabarcoding (eDNA) and macroscopic diet analysis (Macro), depicted as each method individually or pooled (Both).

All predators	Order level			Species level		
	DNA	Macro	Both	DNA	Macro	Both
Number of detections	62.0	19.0	63.0	60.0	17.0	74.0
Additional information	23.0	4.0	25.0	37.0	6.0	42.0
% added	20.9	4.4	22.3	1.2	0.2	1.4
**Badger**						
Number of detections	44.0	15.0	48.0	36.0	10.0	45.0
Additional information	14.0	3.0	16.0	20.0	4.0	24.0
% added	13.9	3.3	15.5	0.7	0.1	0.8
**Fox**						
Number of detections	43.0	14.0	45.0	33.0	7.0	38.0
Additional information	15.0	2.0	16.0	19.0	2.0	20.0
% added	14.7	2.2	15.5	0.6	0.1	0.7
**Martens**						
Number of detections	24.0	9.0	27.0	15.0	7.0	22.0
Additional information	9.0	0.0	9.0	11.0	2.0	13.0
% added	9.4	0.0	9.4	0.4	0.1	0.4

Additional detections and the percentage (%) added to order or species information relative to field observations are reported.

Diet composition was described based on the taxonomically assigned DNA sequences obtained after the quality filtering. For each predator species, we calculated and compared the number of different sequences identified across samples, as well as the number of taxa obtained per sample with each marker. Next, we assessed the taxonomic resolution of diet components by calculating the proportion of sequences identified to order, family, genus and species level, and calculated the frequency of occurrence (FO) of each sequence as the proportion of samples in which a given sequence was found, relative to the total number of samples collected from each predator. We then compared the general extent of omnivory and composition of badger and fox faecal samples by estimating the proportion of samples which comprised one, two or all three diet categories (plants, vertebrates, invertebrates).

The extent of dietary overlap between the two predators was evaluated using non-metric multidimensional scaling (NMDS) by computing the *Jaccard* distance on the presence-absence matrix of faecal samples versus diet components assigned to taxonomic order level, using the *vegan* package v. 2.5-8^44^. The dietary uptake of the two predators was statistically compared by a permutational multivariate analysis of variance on the distance matrices using the *adonis* function in *vegan*.

To assess the effect of faecal age (estimated time of exposure to environmental DNA degradation), we used only badger faecal samples, since the number of fox samples was too low for this analysis. We tested the effect of faecal age on the total number of taxa and reads per sample, using a two-way ANOVA with primer, DNA quality and their interaction as fixed effects. We also analysed the taxonomic resolution of sequence identifications obtained with each of the three universal markers between samples from the two age categories (< 7 days old, > 7 days old, samples more than 4 weeks old were excluded). For this analysis, we used the function *prop.test*, which is a 2-sample test for equality of the proportion of sequences identified to each taxonomic level (order, family, genus and species) between the two sample age categories. The two age categories are meant to serve as a broad separation between relatively “fresh” and “older” samples that are likely to be significantly affected by DNA degradation as a result of the cumulative effect of multiple factors acting during sample exposure to the environment (see also^36,45^).

### Macroscopic diet analysis

Faecal samples were visually investigated, and non-diet surface contamination was removed (*i.e.* invertebrate decomposers or biological matter such as grass). All samples were weighted on a digital scale, transferred into a 20 cm diameter 750 μm sieve, gently rinsed with 500 mL water, and the off-cut water was collected for sedimentation. Sediments (2 mL) were transferred to a petri dish for identification of earthworm cilia following standard procedures^46^. Diet components were separated into 11 dietary categories (*Amphibia*, *Arachnida*, *Aves*, *Diplopoda*, *Gastropoda*, *Insecta*, *Malacostraca*, *Mammalia*, *Oligochaeta*, *Reptilia* and *Plantae*) and identified to the lowest possible taxonomic resolution. Diet components that could not be allocated to any specific category were classified as “Unidentified”.

Mammalian prey were identified to species, genus, family or order level, based on *cuticula*, cell structure, cross section of guard hairs^47^, and teeth, if present in the sample^48^. Birds were determined to taxonomic order level using feathers^49^. Amphibians, reptiles, insects, vegetation and larger undigested body parts of mammalian and bird prey were identified based on field guides and reference collections^48,50,51^. Fruits, berries and cereals were classified based on seed structures using reference collections from native plants and field books with illustrations for comparison. Mammalian bones and tissue without guard hairs or other key-identification traits were assigned to “Mammalian prey”, with no further taxonomic classification. We visually estimated the proportion of each diet category in individual samples, relative to the full volume and weight of each faecal sample. Small stones and host hairs were not included in the diet analysis since these components were classified as “Non-diet”.

### eDNA metabarcoding versus macroscopic diet assessment

To compare the dietary information gathered by use of eDNA metabarcoding versus macroscopic diet analysis, we identified the number of different food components from each method (across all samples within each predator) and compared the taxonomic resolution of the identifications. To evaluate the rate at which new taxa are found within the samples, we generated rarefaction curves of sample richness with 1000 permutations for both methods at all taxonomic levels, using *vegan*.

### Biodiversity assessment and comparison of field observation with dietary analyses

We compiled all existing information on species diversity in the two study areas^37,52,53^, obtained from repeated field surveys by ~ 30 educated specialists starting in 1929 (see Supplementary Table 3 for overview of observed groups, season and timeframe of observations. Note however, that certain taxonomic groups were not surveyed – *e.g.* earthworm).

We compared this aggregated species list with the species information obtained from the two dietary approaches (*i.e.* eDNA metabarcoding and macroscopic analysis). We investigated the taxonomic richness described by each method by assessing the total number of different orders and species detected by each method (diet approaches individually and combined). We then calculated the overall number of matches at species and order level among the three methods (and between the predators), to assess the coverage of each approach in describing the biodiversity in Lille Vildmose. The amount (and lack) of overlap among the three methods was then used as a proxy for understanding whether, and to what extent, the prey diversity obtained by analysing faecal samples from generalist omnivore predators compares to species richness estimates obtained by field observations.

## Results

From April to December 2015, we collected 99 faecal samples for badger, and 72 for fox in Lille Vildmose, Denmark. Samples were approximately evenly distributed among seasons, with a slight overrepresentation during winter, particularly for fox. Overall, badger faecal samples tended to be geographically clustered due to the use of latrines located close to the setts, whereas fox samples were more randomly distributed in the area.

### eDNA metabarcoding diet analysis

eDNA metabarcoding using the three universal markers generated a total of 15,176,073 paired-end sequence reads for which markers and tags could be identified. Our sequence analysis and filtering pipeline discarded 4 (4%) badgers and 1 (1.5%) fox samples (final number of 95 and 71 samples available for dietary analysis, respectively). Number of badger and fox reads varied among samples (mean = 5409, SD = 4822). These reads were used to confirm predator identity. Among the 71 fox samples, we identified 15 samples that contained *Martes*. After applying the filtering pipeline, 13 of these samples did not contain any fox reads, ultimately supporting the assumption that they might be faeces deposited by marten rather than fox. The remaining two samples with marten sequences contained both fox and marten, and their origin could not be ascertained. These two samples were excluded from further analyses. Among the 95 badger samples, we identified one that contained *Vulpes* sequences and no badger sequences. This sample was included in the analysis for the fox, with a final dataset of 57 fox samples available for analysis. Due to low sample size, we excluded the 13 marten samples from statistical analysis of diet comparison, but reported on the composition of these samples separately, and included the dietary information in the analysis of applicability of dietary approaches in biodiversity assessment.

**Figure 2:**
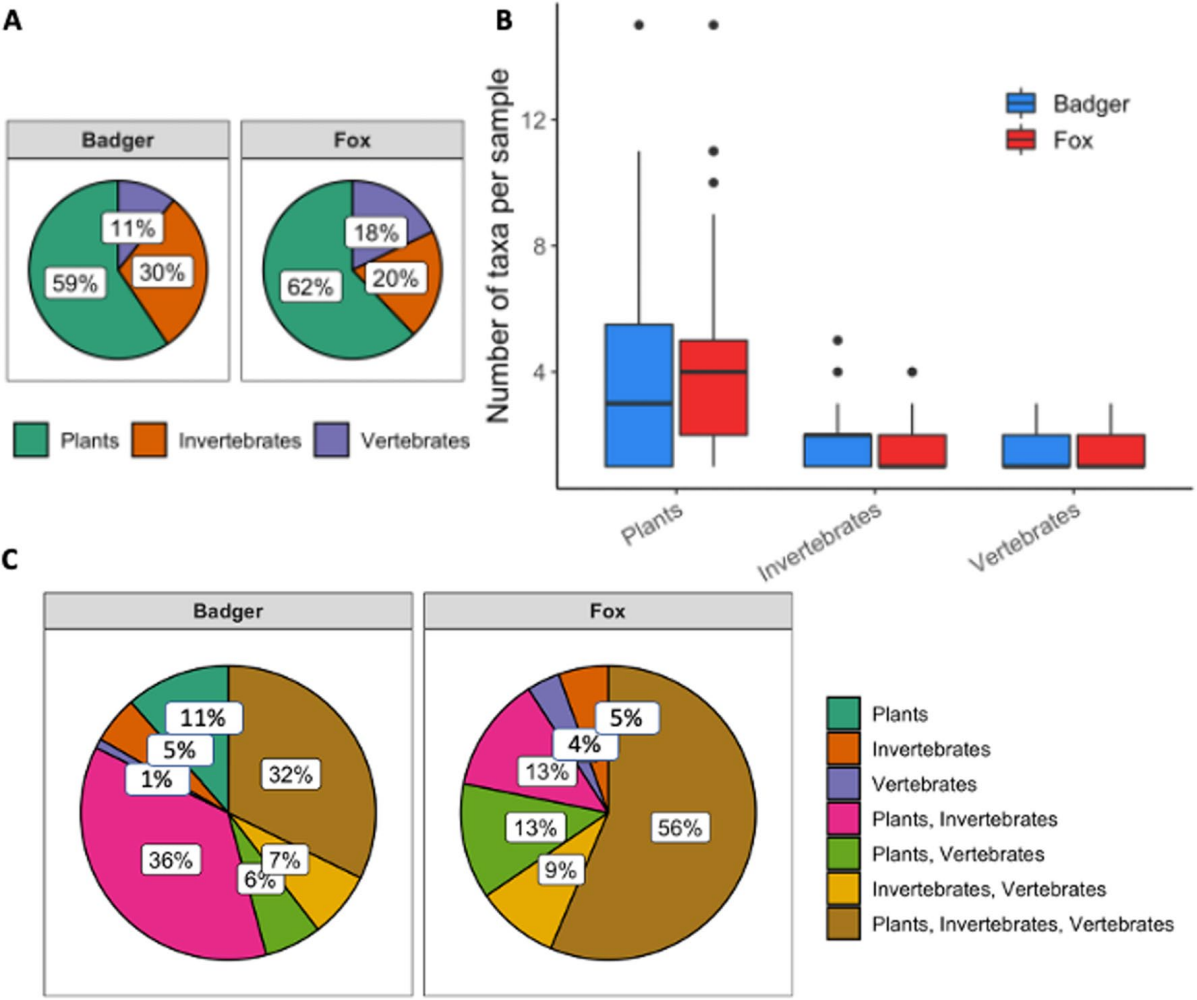
Prey diversity observed in the diet of badger and fox using eDNA metabarcoding. (**A**) proportion of different sequences identified with each of the three markers in badger and fox samples, (**B**) number of different taxa per sample, (**C**) proportion of samples that contained a prey identified with a single, two, or all three universal markers targeting plant, invertebrate and vertebrate DNA in badger and fox faecal samples.

We identified 91 and 87 plant, 14 and 17 vertebrate, and 53 and 29 invertebrate sequences in badger and fox samples, respectively (Fig. 2A). Sequences were identified to taxa at different taxonomic levels (Supplementary Table 4), and the taxonomic resolution of the three universal markers differed considerably. Taxonomic resolution was higher for vertebrates (33% and 43% of sequences identified to species level) compared to plants (7% and 8%) and invertebrates (20% and 13%) in badger and fox samples, respectively. Using the invertebrate marker, 32% of the sequences in badger, and 18% fox samples were only identified to taxonomic levels higher than order.

The number of different taxa identified per sample, derived by pooling multiple sequences identified to the same taxa within each sample, differed between markers and was greater for plants in both predators (Fig. 2B). Multiple different plant taxa were found in scats from both predators, while the invertebrate and vertebrate components of the diet were less diverse. Within the plant-based components of the diet, the sequences assigned to the orders *Fagales*, *Poales*, *Rosales* and *Caryophylales* had the highest FO in scats from both badger and fox (Supplementary Table 4). Concerning the vertebrate diet components, *Artiodactyla* had high FO (mostly in fox diet). Among taxa identified with the invertebrate marker, *Coleoptera* were most frequently consumed by badger, while *Lepidoptera* were mostly found in fox diet (Supplementary Table 4). Most diet components occurred with a low frequency, but with some noticeable differences between the two predators (e.g. *Quercus* 16% FO in the badger and only 2% in the fox, and *Sus scrofa* with FO 39% in fox and only 3% in badger), and with some taxon exclusively detected in scats of one predator (e.g. *Anseriformes* which occurred only in one fox sample).

**Figure 3:**
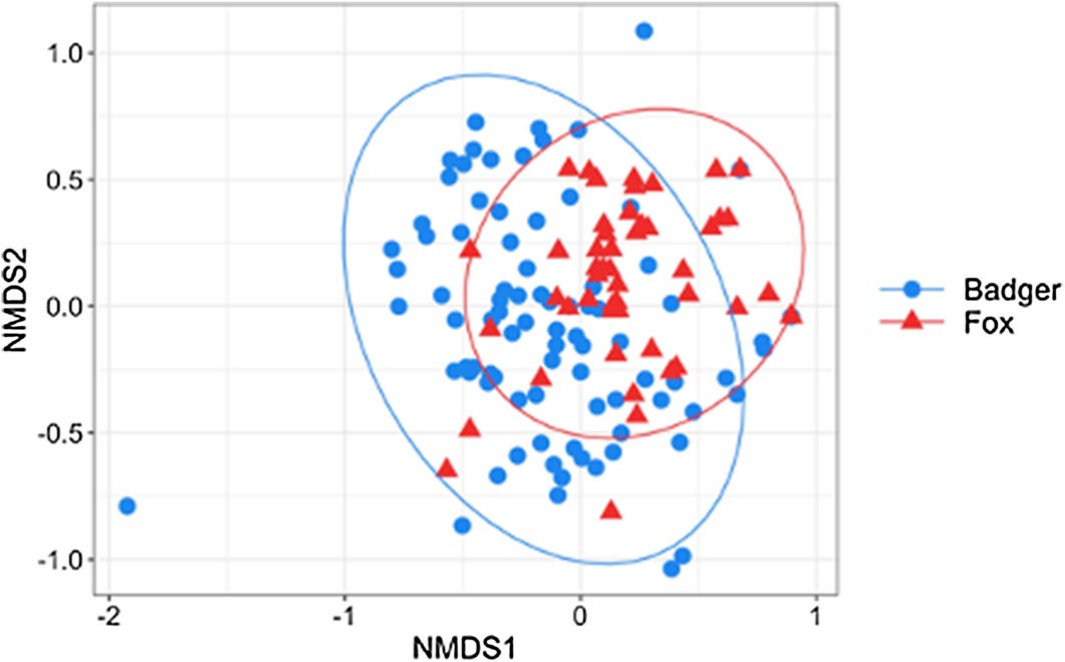
Dietary overlap between fox and badger, analysed by NMDS. Points represent individual samples with 95% confidence ellipses for badger (blue circles) and fox (red triangles). Using *adonis,* we identified significant dietary divergence between the two predators (df = 1, F = 8.82, *p* = 0.01).

To assess the general composition of badger and fox diet, we calculated the proportion of samples that contained prey components detected with one, two or three universal markers (Fig. 2C). Fox samples more commonly (56% *vs.* 32%) contained all three food categories, whereas an exclusively plant-based composition was found only in badger faeces (11%). These differences were additionally supported by the NMDS results (Fig. 3) that, despite a considerable overlap, showed a significant dietary differentiation between the two predators (d.f. = 1, F = 8.71, *p* = 0.01). For this analysis, we had to exclude the outlier fox sample that contained *Anseriformes,* which was masking the overall interpretation of dietary variation between badger and fox.

**Figure 4:**
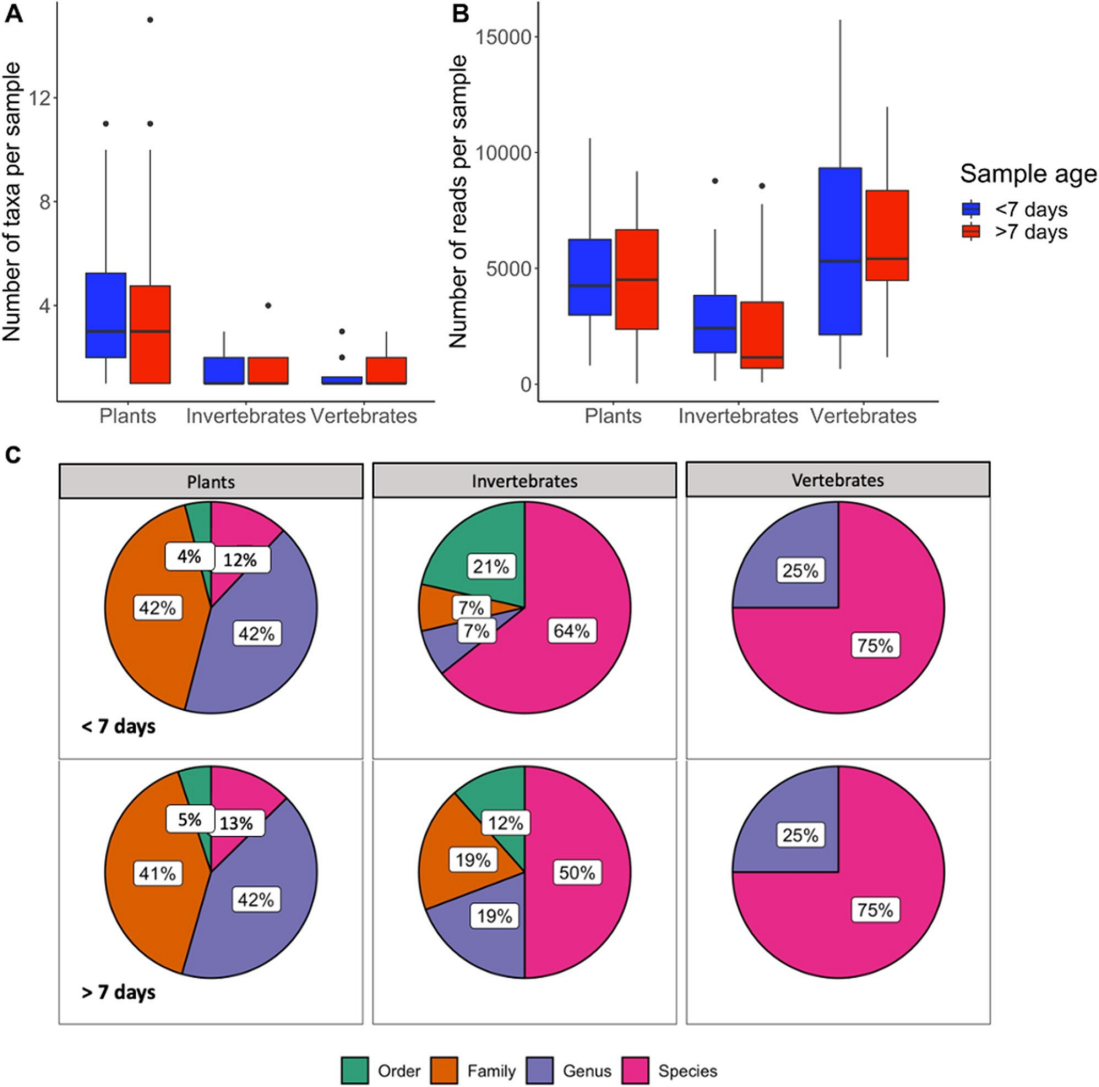
The impact of DNA quality (time exposed to environmental DNA degradation) on (**A**) number of taxa, (**B**) number of sequence reads obtained per sample, (**C**) the proportion of different sequences identified to order, family, genus and species using the three universal markers.

Using badger samples (N = 95), we found no significant effect of faecal age on the number of taxa detected per sample (F_1,169_ = 0.033, *p* = 0.855, Fig. 4A), or on the number of reads obtained per sample (F_1,169_ = 0.019, *p* = 0.913, Fig. 4B). A significant effect of the marker was found in both analyses (number of taxa: F_2,169_ = 9.06, *p* < 0.001; number of reads: F_2,169_ = 9.60, *p* < 0.001), but no interaction effect. An analysis of the taxonomic resolution of sequences assignation likewise did not indicate any effect of sample age (*p* < 0.5 for all comparisons between the two age categories), although species level identifications increased 14% for invertebrates when fresher samples were used (Fig. 4C, d.f. = 1, X^2^ = 0.29, 95% CI = − 0.23–0.52, *p* = 0.6).

Among the 13 marten samples, 40%, 33% and 27% of all sequences were attributed to plant, invertebrate and vertebrate diet components, respectively (note that blocking primers for *Martes* where not employed, which most likely affected detection efficiency of vertebrates). We found an average of 6 sequences per sample, although with large variation (i.e. 2 samples with 12 and 13 sequences, and a few samples with only 2 sequences). Overall, 35% of those sequences were identified to species level, 26% to genus, 24% to family, and finally, 6% to order, and 9% to higher taxonomic levels (Supplementary Table 4).

## Macroscopic diet analysis

Using the macroscopic diet identification technique, 61% and 58% of all dietary identifications were classified to class level, 20% and 13% to order level, 3% and 16% to family, 10% and 7% to genus, and only 5% and 5% to species level, in badger and fox samples, respectively. Overall, invertebrate and plant material were the most frequently occurring diet components in badger samples (43% and 30% occurrences), whereas vertebrate and plant material were predominant in fox (54% and 28%, respectively) (Supplementary Table 5 and Fig. S1). Importantly, macroscopic diet analysis revealed the presence of earthworms in 86% of badger samples, but none in fox samples.

Similarly, in the marten samples, 53%, 14%, 7%, 8% and 18% of the dietary identifications were classified to class, order, family, genus and species, respectively. Marten samples most frequently contained vertebrate material (57%) followed by plant material (29%) and invertebrate diet components (12%), and no earthworm were identified in those samples (Supplementary Table 5 and Fig. S1).

### eDNA metabarcoding versus classic macroscopic diet assessment

**Figure 5:**
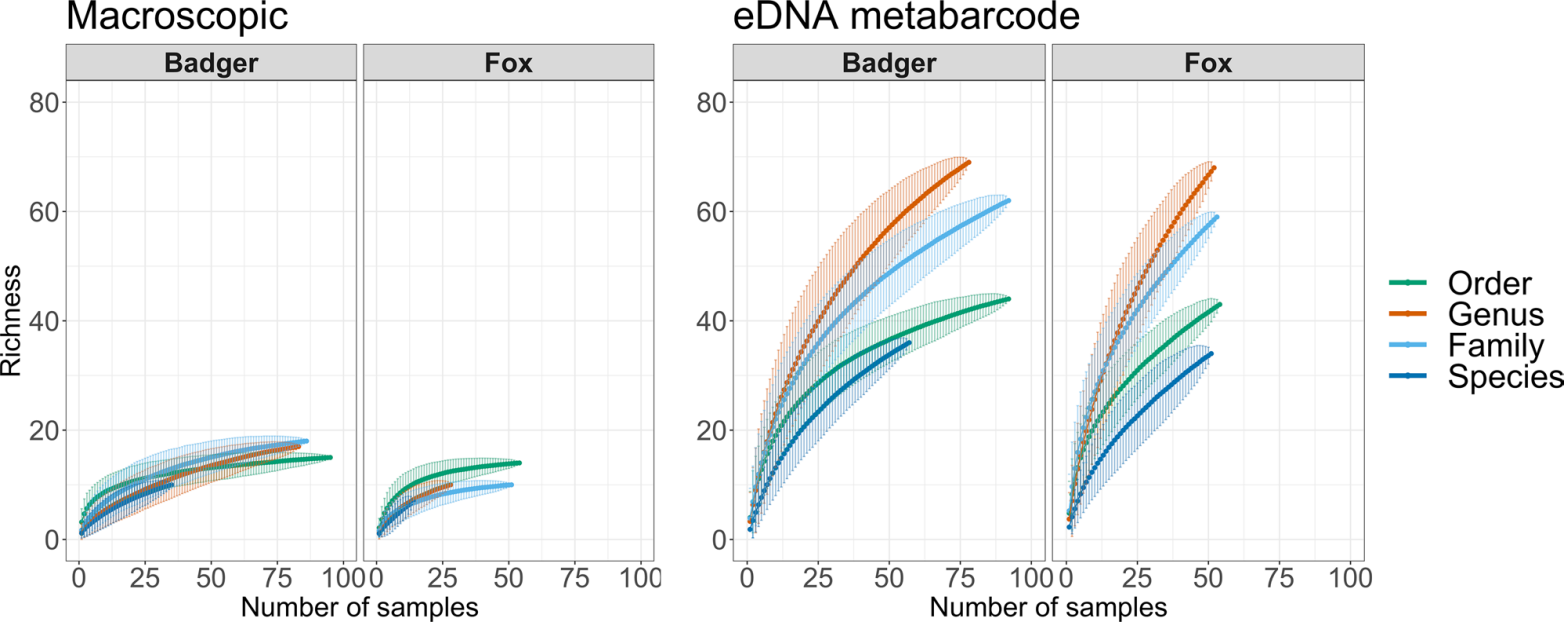
Rarefaction curves (1000 permutations) with Mao Tau values estimating species accumulation at different taxonomic levels for number of taxa in badger and fox separately; left panel = macroscopic diet analysis, right panel = eDNA metabarcoding.

Overall, we found that eDNA metabarcoding led to higher taxonomic resolution and higher prey diversity, compared to the macroscopic diet analysis, and this pattern was consistent for both fox and badger (Fig. S2). When assessing the number of diet components identified to the lowest possible taxonomic level, eDNA metabarcoding consistently outperformed the macroscopic analysis, especially at the genus and species levels (Fig. S2). Similarly, rarefaction curves (Fig. 5) showed that eDNA metabarcoding consistently resulted in higher richness and the curves did not level off.

### Biodiversity assessment and comparison of field observation with dietary analyses

We compared the list of species observed in the field (observation data) to eDNA metabarcoding and macroscopic diet analyses, to assess whether dietary information from non-invasively collected faecal samples from generalist predators can be used in biodiversity assessments. In this comparison, we included the 13 marten samples, since this species is common in Denmark and may contribute to biodiversity estimates derived from faecal analysis.

We found that the addition of dietary information to field observations resulted in a noteworthy increase in the number of different detections at the order level (22% increase when combining diet analysis and field observations, Table 1). However, when analysing biodiversity at the species level, the addition of prey species derived from dietary analysis did not significantly improve the number of species detected (an increase of 1.4%). Overall, the eDNA metabarcoding approach improved biodiversity assessment the most, when combined with field observations. eDNA methods identified 20% additional orders and 1.2% additional species as compared to 4% additional orders and 0.2% and additional species by macroscopic analysis. Finally, we found that the combination of all methods (field observations, eDNA metabarcoding and macroscopic identification) generated the best coverage (highest richness) of orders present in the area (Table 1 and Supplementary Fig. S3).

Importantly, the two diet identification approaches revealed information about orders and species that went unnoticed during field observations. In fact, diet identifications led to the detection of 42 species and 25 orders, which were not observed in the field survey (Table 1 and Supplementary Table 6). Six out of the 42 species (and one order without species identification; *Zingiberales*) were most likely not present in the study area. However, for all these species, either the genus or family was likely to be present in the sampling area, suggesting that the lack of an appropriate reference in the sequence database might have led to the identification of an incorrect, but closely related, species. Additionally, 13 of the prey species not recorded by field observations might represent later introduction as edible plants (*e.g.* the American cranberry, *Vaccinium macrocarpon*) or invasive species (*e.g.* the alien moss, *Orthodontium lineare*). However, for many of the potentially introduced species, limited information on distribution is available and it was therefore not possible to assess whether these detections were legitimate (Supplementary Table 6).

## Discussion

A number of studies indicate that molecular methods cannot completely replace traditional macroscopic diet analyses^54,55^, but there is a wide agreement that there is much to be gained by using metabarcoding to qualitatively assess diet composition compared to macroscopic analysis, including higher taxonomic precision, sensitivity, and cost-efficiency^54^. In this study, we found that eDNA metabarcoding applied to faecal samples of red fox and Eurasian badger complemented and refined results of macroscopic diet analysis, improving detection and taxonomic resolution. In addition, our results suggest that dietary analysis of omnivore predators can improve information about habitat specific biodiversity, particularly when methods are combined. In accordance to other recent eDNA studies^56^, we also found that predator faeces can be misidentified by field collectors, and that molecular tools are essential to ascertain the source of the faecal sample (*i.e.* fox and marten faecal remains are morphologically very similar). Using only macroscopic diet analysis, could in this sense, cause incorrect diet determination, if including samples from unintended predators.

Diet analyses can provide information not only on food preferences of individuals and their fitness level^57^, but can also contribute to the description of biodiversity in the foraging area, especially in the case of generalist omnivores^30,31^. However, results on diet composition can be influenced by the detection probabilities of different diet components connected to the chosen methodology, *i.e.* macroscopic *vs.* molecular^34,35^. The main food categories detected with both molecular and macroscopic approaches in this study showed a good level of concordance with previous macroscopic studies conducted in Denmark^58–60^, but the eDNA metabarcoding approach retrieved a larger number of dietary components and with higher taxonomic resolution than the macroscopic analysis. Such finding emphasises the usefulness of eDNA in dietary analysis, but also flags that differences exist in the bias associated with various diet identification methods.

Both fox and badger are considered omnivores, but with a larger proportion of the diet constituted by protein sources^57,61^. Our observations are in overall agreement with this expectation, with the majority of fox samples including a combination of all food components. For badger faeces, we likewise report a large proportion of samples with all three major food components present, but they were preferentially constituted by a combination of plant and invertebrate sequences, highlighting that, although partly overlapping, the diet of these two species is somewhat differentiated (Fig. 2 and Fig. S1). However, our eDNA results showed a large number of sequences identifying plant material for both omnivores, an observation confirmed by macroscopic analysis for badger but not for fox. This deviation from the expected diet can be partly explained by the plasticity of the species^57^, and by the fact that we combined data collected during seasons with different productivity levels which might induce temporal deviations from the preferred food intake^57,61^. In this context, a larger sample size from each season would allow inferences on temporal variation in diet composition of both predators within the study area and provide more accurate estimates of biodiversity. In addition, with the eDNA approach, we did not employ markers targeting *oligochaete*, an important food component for the badger, inducing differences in the dietary conclusions reached using macroscopic *vs.* eDNA metabarcoding.

Another notable difference between eDNA metabarcoding and macroscopic results is the differential detectability of some taxa. For example, gastropods and, possibly, amphibians are more difficult to identify with macroscopic analysis since they are mostly constituted by soft parts that are easily degraded or become unrecognisable during the digestion process, while insects are more easily identified with this method due to their exoskeleton that assures the survival of diagnostics characteristics. In contrast, eDNA metabarcoding might be able to identify diet components that leave no morphologically recognisable remains in the faecal sample, and might even be able to pick up the signal of prey components present as traces^11^. Rare and accidentally ingested food components are expected to occur at low frequencies in the samples and may contribute to increased dietary diversity estimates by the eDNA metabarcoding approach, as supported by the rarefaction curves. This also suggests that higher sample sizes could potentially further increase the number of taxa detected with eDNA metabarcoding, particularly at genus and family level. In contrast, macroscopic identification revealed overall lower sample richness, regardless of the taxonomic level in focus. While this aspect has to be accounted for when interpreting eDNA metabarcoding results for diet assessments, it has useful implications in the context of biodiversity surveys. However, taxa detectability varies in metabarcoding approaches as well, due to a number of widely acknowledged biological and technical reasons^45,62^ that should be considered when designing a study and drawing inferences.

Interestingly, and in contrast to previously published expectations^36,45^, detection performance (number of taxa and reads obtained) using the eDNA metabarcoding approach was not influenced by the age of the sample, confirming robustness of the markers and efficacy of the sequence filtering protocols applied. However, we observed differences in the taxonomic resolution in relation to the age of our samples, which was not directly tested in the above-mentioned studies. We found that resolution was unchanged among identifications with the plant and vertebrate marker, but species level assignations decreased considerably, although not with statistical significance, for the invertebrate marker in older samples. In addition, with the invertebrate marker, we observed an increase in family identifications and a reduction in order attributions with increasing age of the sample (Fig. 4C). This may be due to differences in the sample composition, but we cannot exclude possible contamination due to colonization of the sample by insects over time prior to collection. Thus, given the number of ecological and temporal variables that are likely to influence these results, we recommend collection of fresh samples for eDNA metabarcoding diet analyses.

Our study, revealing high performance of eDNA metabarcoding and its complementarity to macroscopic analyses, adds to previous research supporting the integration of dietary approaches in biodiversity surveys and monitoring^31,35,63^. Indeed, eDNA based methods are increasingly applied in monitoring of vertebrates and invertebrates from water or soil samples (see^13,64,65^). Our approach demonstrates an additional application of eDNA metabarcoding in the monitoring of biodiversity by use of non-invasively collected faecal samples from local omnivorous predators.

Field observations from the sampling area revealed an overall higher species and order diversity, which was not unexpected since field observation data were collected over a long timeframe (regular surveys between 1929 and 2016, see Supplementary Table 3), combined with the fact that generalist predators do not feed on all available prey components in a habitat, but try to match their macronutrients requirements by minimal effort. Additionally, prey detected with the dietary approaches may be locally clustered with regard to the two predators foraging habitat. Prey availability and accessibility^57,61^, and the presence of other potential competing species could also influence dietary preferences. However, our diet analyses detected an additional 25 orders (and 42 species), which went undetected by field observations. This suggests that dietary studies provide the opportunity to identify rare and potentially novel species in a given study area, depending on the dietary preferences of the predator studied. It should be noted that among the two dietary approaches, the eDNA metabarcoding approach led to the largest number of additional orders (23), while the macroscopic analysis identified 4 additional orders which were not identified by field surveys nor eDNA metabarcoding. While some of the taxa identified by the eDNA metabarcoding only might be attributed to misassignments due to the absence of an appropriate reference sequence in the EMBL database, others might result from recent introductions since the last survey, like in the case of edible or horticultural plants, or natural invasions, such as alien moss or coleoptera and gastropod species.

Our order-level analysis furthermore suggests that certain components of biodiversity, and therefore their associated ecosystem function, could be systematically underestimated by field surveys. A number of reasons may explain this pattern. First, common species, such as slugs or common house fly, may not be noted during field surveys due to their common occurrence (and, thereby potentially not considered important for biodiversity). Next, rare species in low population density, or elusive species, may be difficult to detect, even if surveys are repeated over time. Indeed, the use of local predators as sampling assistants may thereby aid the compilation of species inventories, as supported by the observation that both dietary approaches identified a number of species which may present cryptic behaviours. For example, insect and lice species that live in bark, and slugs that are mostly active during and after heavy rain or live underground, were detected using eDNA metabarcoding, but absent in field observations. Detection of such species in the field may be particularly cumbersome and require invasive sampling, which is adverted by use of “sampling assistants” in the form of faecal material deposited by local omnivore predators. While dietary approaches cannot substitute field observations entirely for biodiversity assessments, due to the limitations described above, their integration with other methods would likely improve our current understanding of community composition. Particularly, the bias associated with each of the applied methods differs broadly, and complementing these methods thereby paints a much more robust picture of biodiversity within a given study area.

To compensate for biases caused by the predators’ preferences, behaviour, and individual choices in terms of food selection, habitat use and activity patterns, the combination of multiple predator species, preferably showing only partially overlapping food preferences, could be applied like in the present study where we included faecal samples from badger, fox and marten. In addition, compared to observational studies typically requiring long time periods for implementation, diet analysis, particularly through eDNA metabarcoding, can be efficiently performed on large samples sizes from a single sampling event and can be repeated over time^12^. This would allow timely detection of changes in prey community composition that could be related to prey distributional shifts as a consequence of climate modifications or human exploitation and invasive species. eDNA metabarcoding diet analysis of selected generalist predators, utilizing appropriate sampling strategies and relevant markers covering the full diet spectrum, can effectively contribute to biodiversity monitoring and provide timely information necessary to cope with rapid anthropogenically driven environmental changes.

## Supplementary Information

Supplementary Information.

## Data Availability

Data from macroscopic and eDNA metabarcode is available from Figshare at 10.6084/m9.figshare.14182817.
