# Author Correction: eDNA metabarcoding for biodiversity assessment, generalist predators as sampling assistants

**DOI:** 10.1038/s41598-021-96412-6

**Published:** 2021-08-16

**Authors:** Louise Nørgaard, Carsten Riis Olesen, Kristian Trøjelsgaard, Cino Pertoldi, Jeppe Lund Nielsen, Pierre Taberlet, Aritz Ruiz-González, Marta De Barba, Laura Iacolina

**Affiliations:** 1 Department of Chemistry and Bioscience, Aalborg University, Fredrik Bajers Vej 7H, 9220 Aalborg, Denmark; 2 School of Biological Sciences, Monash University, 18 Innovation Walk, Melbourne, 3800 Australia; 3 Danmarks Jægerforbund, Molsvej 34, 8410 Rønde, Denmark; 4 Aalborg Zoo, Mølleparkvej 63, 9000 Aalborg, Denmark; 5 CNRS, Laboratoire D’Ecologie Alpine (LECA), Univ. Grenoble Alpes, 38000 Grenoble, France; 6 UiT – The Arctic University of Norway, Tromsø Museum, Hansine Hansens veg 18, 9019 Tromsö, Norway; 7 Department of Zoology and Animal Cell Biology, University of the Basque Country UPV/EHU, C/Paseo de la Universidad 7, 01006 Vitoria-Gasteiz, Spain; 8 Lascaray Research Center, University of the Basque Country (UPV/EHU), Avda. Miguel de Unamuno, 3, 01006 Vitoria-Gasteiz, Spain; 9 Department of Biodiversity, University of Primorska, Glagoljaška 8, 6000 Koper, Slovenia

Correction to: *Scientific Reports* 10.1038/s41598-021-85488-9, published online 25 March 2021

The original version of this Article contained an error in the Acknowledgments section.

“We thank biologists involved in data collection and Delphine Rioux for help in the laboratory. This project was funded by Aage V. Jensen Naturfond, 15. Juni Fonden, and Aalborg Zoo Conservation Foundation (AZCF, Grant Number 3-2017). LI received funding from the Horizon 2020 research and innovation programme under the Marie Sklodowska-Curie Action (Grant Agreement No. 656697).”

now reads:

“We thank biologists involved in data collection and Delphine Rioux for help in the laboratory. This project was funded by Aage V. Jensen Naturfond, 15. Juni Fonden, Jægernes Naturfond, and Aalborg Zoo Conservation Foundation (AZCF, Grant Number 3-2017). LI received funding from the Horizon 2020 research and innovation programme under the Marie Sklodowska-Curie Action (Grant Agreement No. 656697).”

The original Article has been corrected.

