# Supplementary material for: eDNA metabarcoding for biodiversity assessment, generalist predators as sampling assistants
Source: Sci Rep. 2021 Mar 25;11:6820. doi: 10.1038/s41598-021-85488-9 (PMC7994446; doi:10.1038/s41598-021-85488-9)
Supplement: Supplementary file 1 — Supplementary Information. [file 41598_2021_85488_MOESM1_ESM.pdf]

eDNA metabarcoding for biodiversity assessment, generalist predators as sampling assistants

Louise Nørgaard[1,2*], Carsten Riis Olesen[3], Kristian Trøjelsgaard[1], Cino Pertoldi[1,4], Jeppe Lund Nielsen[1], Pierre Taberlet[5,6], Aritz Ruiz-González[,8], Marta De Barba[5°] & Laura Iacolina[1,4,9°]

Supplementary material

Table 1. Blocking oligonucleotides developed to prevent amplification of host DNA (European badger and red fox) with the vertebrate marker V5.

Target species	Primer name	Primer sequence 5'-3'
European badger	Meles_V5_B	ACTATGCTCAGCCCTAAACATAGATAATTCATAGAAC-C3
Red fox	Vulpes_V5_F	ACTATGCTTAGCCCTAAACATAAATAGTTCTATAACA-C3

Table 2. DNA concentration of taxa used to prepare the positive control DNA samples and number of positive controls where the taxon was detected (out of 10). Each DNA sample was diluted 1:100 in the positive control.

Taxa	Initial DNA concentration ng/ul	Number of positive controls where the taxon was detected
**Plants**		
Carramboa trugillensis	10.80	9
Libanothamnus pluvulus	5.14	9
Passiflora crenata	22.00	9
**Vertebrates**		
Ursus arctos	0.23	0
Cephalophus monticola	9.26	9
Pagophyla eburnea	3.75	0
**Invertebrates**		
Coleoptera	7.88	9
Orthoptera	8.38	0
Mantodea	3.75	4

Supplementary note 1. eDNA metabarcoding

DNA extraction and amplification procedure:

DNA extraction from collected subsamples was performed following the protocol described in Taberlet et al. (2012), using approximately 1-2 g of dried faeces and 5-10 mL of phosphate buffer (Na_2HPO_4; 0.12 M; pH ~8) per sample, and recovering the DNA extracts in 100 µL. Faecal DNA extractions were carried out in a room dedicated to processing eDNA samples. Ten extraction negative controls (containing only extraction reagents and no faecal material) were included to monitor contamination and were processed following the same protocol as used for the faecal samples. Diet analysis was performed by co-amplifying three universal markers, targeting short (<100 bp) and variable DNA fragments of components of the diet: (i) plants (on the P6 loop of the chloroplast trnL (UAA) intron), GH in[1] or Sper01 in[2], (ii) vertebrate (marker V5, V5 loop of the mitochondrial 12S gene in[3] or Vert01 in[2], and (iii) invertebrate (on the mitochondrial 16S gene, MAV in[4] close to Arth01 and Moll01 in[2]. For simplicity, throughout the paper, universal markers will be referred to as the target diet categories i.e. plants, invertebrates and vertebrates. A blocking oligonucleotide was designed and used to avoid amplification with the vertebrate marker of host DNA from each of the two omnivorous predators investigated (Supplementary Table 1). In addition, blocking oligonucleotides specific to human and mammalian sequences were included to prevent their amplification with the vertebrate and invertebrate universal markers, respectively, as suggested in De Barba *et al.*[4].

Amplifications were carried out using the Qiagen Multiplex PCR kit (QIAgen GmbH) and contained 1x Qiagen Master Mix, 0.5x Qiagen Q Solution, 0.1 µM F/R universal markers, 1 µM of each blocking marker and 2 µL DNA extract, in 20 µL reaction volume. Thermocycling conditions had an initial denaturation step of 15 minutes at 95°C, followed by 45 cycles of 30 seconds at 94°C, 90 seconds at 55°C, 60 seconds at 72°C, and a final elongation of 10 minutes at 60°C. Following the protocol in De Barba *et al.* (2014), we used PCR negative (n=10) and positive (n=10) controls to monitor the performance of the amplification and sequencing, and to guide the selection of filtering parameters in the sequence analysis process. Positive controls were made by mixing known quantities of the DNA extract of nine taxa representing plants, vertebrates and invertebrates (Supplementary Table 2). Three PCR replicates were performed for all samples. Markers used in each PCR were uniquely modified by the addition of molecular identifier tags on the 5' end, to allow bioinformatic assignment of sequence reads to their source samples. Tags differed on both

ends of a PCR product and were composed by eight nucleotides, containing at least five differences among them[5]. Empty PCR wells, corresponding to unused tag combinations, were included in the experiment to control for potential tag-jumping events[6]. PCR products were purified using the MinElute PCR purification kit (QIAgen GmbH), and finally mixed together in equivolume proportions. The sequencing was carried out on HiSeq 2500 platform (Illumina Inc., San Diego, CA, USA), following the manufacturer's instructions, using a commercial service (www.fasteris.com).

Sequence analysis and filtering protocol (modified from De Barba et al. 2014):

The sequence reads were first analysed using the OBITools package[7]. Direct and reverse reads corresponding to a single molecule were assembled and primers and tags were identified. The amplified regions, excluding primers and tags, were kept for further analysis. A separate dataset was created for each marker and strictly identical sequences were clustered together, keeping the information about their distributions among samples. Sequences shorter than 10 (plants, invertebrates) or 30 (vertebrates) bp, or with occurrence lower than 1000 were excluded. Each sequence within a PCR product was classified into the categories of 'head' (the most common sequence within a group of sequences differing by a single indel/substitution), 'internal' (sequences less frequent within the group of related sequences, *i.e.* corresponding to amplification/sequencing errors) or 'singleton' (a sequence with no other variant differing by a single indel/substitution). Taxon assignation was achieved by finding highly similar sequences to the query sequence in the reference databases and assigning a unique taxon to each sequence.

Taxonomically assigned sequences of each marker were then further filtered using R v. 3.3.3[8]. We used the positive and negative controls included in the experiment to set filtering parameters and to evaluate the performance of the experiment. Initially, we discarded sequences with a per sample frequency of occurrence below a given threshold, that was set separately based on each marker data (<0.01 for plants, <0.05 for vertebrates, and <0.02 for invertebrates). Sequences not identified as "head" in ≥2, or "singleton" in 3 replicates in at least one sample were considered erroneous and therefore deleted. The sequence data of PCR replicates of every sample were then compared to evaluate the repeatability of results and identify unreliable amplifications. First, we compared PCR replicates of each sample based on sequence frequency of occurrence. A matrix of pairwise distances among sample replicates was created by computing *Rekonen Similarity Index*[9] based on sequence frequency data. Distance measures took a minimum value of 0 if the sets of relative abundances

compared were identical, and a maximum of 1 for completely distinct assemblages (no sequences in common). Only samples having at least 2 out of 3 replicates with distance among each other < 0.6 (threshold set by comparing replicate similarity of PCR negative and positive controls, and faecal samples) were kept for further analysis. Secondly, we compared sequence presence/absence among sample replicates and requested a given sequence to be observed in at least 2 replicates of a sample. Sample replicates were finally combined to obtain a consensus sequence profile for each sample, by taking the mean of the non-zero sequence counts of sample replicates. PCR negative control consensus profiles were inspected and compared to those of positive controls and faecal samples to identify sequences deriving from contamination and low quantity DNA samples more likely to produce unreliable results. Finally, to increase accuracy of the automatic taxonomic assignation, we considered only sequences with identity ≥90% over the entire query sequence length with any reference sequence.

Table 3. Overview of species groups detected in the two study areas, Tofte Skov and Høstemark in Lille Vildmose by periodic field observations from 1929-2016. Generally, insects and spiders, as well as most vascular plants, were identified during the growing season (spring to fall; May - September), whereas mammals and birds were subject to more prolonged periods of study across all seasons.

**Species group**	**Period of observation**		**Season of observation**	**Order**	**Families**
	Tofte Skov	Høstemark			
**Plants**					
Vascular plants	2003-2009	2003-2009	Spring-Fall	Multiple	Multiple
Lichens	2016	1993-2000	Spring-Fall	Multiple	Multiple
Mosses	2001-2012	1994-2000	Spring-Fall	Multiple	Multiple
Mushrooms	2016	1992-1994	Spring-Fall	Multiple	Multiple
**Invertebrates**					
Beetles	2003-2010	1986-2000	Summer	Coleoptera	Multiple
Spiders	2003-2010		Summer	Araneae	Multiple
Dragon- and damselflies		1994-1998	Summer	Odonata	Multiple
Ants*		1994-2000	Summer	Hymenoptera	Formicidae
Butterflies		1922-2000	Summer	Lepidoptera	(Multiple
Buzz flies		1974-2000	Summer	Diptera	Syrphidae
Shield bugs		1988-2000	Summer	Hemiptera	(Multiple
**Vertebrates**					
Birds	1929-2010	1929-2010	All year	Multiple	Multiple
Mammals	1945-2010	1945-2010	All year	Multiple	Multiple
Reptiles and amphibians		1945-2010	All year	Multiple	Multiple

Table 4. Taxa identified in badger, fox and marten faecal samples using eDNA metabarcoding. Frequency of Occurrence (FO) is calculated for each sequence (badger (B), N = 96; fox (F), N = 56; martens (M), N = 13).

Sequence	Order	Family	Genus	Species	B	F	M
**gh_00095**	*Apiales*	*Apiaceae*	*Aegopodium*	*Aegopodium podagraria*		1.8	
**gh_00029**	*Apiales*	*Apiaceae*	*Azorella*	*Azorella caespitosa*	5.2	3.6	7.7
**gh_00029**	*Apiales*	*Apiaceae*	*Azorella*	*Azorella caespitosa*			
**gh_00029**	*Apiales*	*Apiaceae*	*Azorella*	*Azorella caespitosa*			
**gh_00064**	*Apiales*	*Apiaceae*				1.8	7.7
**gh_00307**	*Asparagales*	*Asparagaceae*	*Maianthemum*		2.1		
**gh_00163**	*Asparagales*	*Iridaceae*	*Iris*			1.8	
**gh_00089**	*Asterales*	*Asteraceae*	*Achillea*		5.2		
**gh_00033**	*Asterales*	*Asteraceae*					
**gh_00033**	*Asterales*	*Asteraceae*			2.1	5.4	
**gh_00042**	*Asterales*	*Asteraceae*			3.1	3.6	
**gh_00042**	*Asterales*	*Asteraceae*					
**gh_00074**	*Asterales*	*Asteraceae*			3.1		
**gh_00085**	*Asterales*	*Asteraceae*				5.4	
**gh_00097**	*Asterales*	*Asteraceae*			2.1		
**gh_00112**	*Asterales*	*Asteraceae*			1.0		
**gh_00041**	*Asterales*	*Asteraceae*	*Scorzoneroides*				
**gh_00041**	*Asterales*	*Asteraceae*	*Scorzoneroides*		3.1	7.1	
**gh_00006**	*Asterales*				7.3	10.7	7.7
**gh_00006**	*Asterales*						
**gh_00006**	*Asterales*						
**gh_00079**	*Asterales*					1.8	
**gh_00301**	*Asterales*					1.8	
**gh_00113**	*Boraginales*	*Hydrophyllaceae*	*Phacelia*	*Phacelia tanacetifolia*		1.8	
**gh_00160**	*Brassicales*	*Brassicaceae*			1.0		
**gh_00122**	*Brassicales*	*Caricaceae*			1.0		
**gh_00086**	*Caryophyllales*	*Caryophyllaceae*	*Cerastium*		1.0	5.4	
**gh_00086**	*Caryophyllales*	*Caryophyllaceae*	*Cerastium*				
**gh_00044**	*Caryophyllales*	*Caryophyllaceae*			3.1	3.6	
**gh_00044**	*Caryophyllales*	*Caryophyllaceae*					
**gh_00055**	*Caryophyllales*	*Caryophyllaceae*			5.2	7.1	
**gh_00055**	*Caryophyllales*	*Caryophyllaceae*					
**gh_00073**	*Caryophyllales*	*Caryophyllaceae*			2.1		
**gh_00233**	*Caryophyllales*	*Caryophyllaceae*	*Silene*		1.0		
**gh_00194**	*Caryophyllales*	*Caryophyllaceae*	*Stellaria*		1.0	3.6	
**gh_00194**	*Caryophyllales*	*Caryophyllaceae*	*Stellaria*				
**gh_00004**	*Caryophyllales*	*Chenopodiaceae*	*Beta*	*Beta vulgaris*		16.1	
**gh_00111**	*Caryophyllales*	*Polygonaceae*			1.0	3.6	
**gh_00111**	*Caryophyllales*	*Polygonaceae*					
**gh_00143**	*Caryophyllales*	*Polygonaceae*				1.8	
**gh_00148**	*Caryophyllales*	*Polygonaceae*			2.1		
**gh_00030**	*Caryophyllales*	*Polygonaceae*	*Rumex*		8.3	8.9	7.7
**gh_00030**	*Caryophyllales*	*Polygonaceae*	*Rumex*				
**gh_00030**	*Caryophyllales*	*Polygonaceae*	*Rumex*				

**gh_00104**	*Cucurbitales*	*Cucurbitaceae*			1.0	3.6	
**gh_00104**	*Cucurbitales*	*Cucurbitaceae*					
**gh_00087**	*Cupressales*	*Cupressaceae*					7.7
**gh_00266**	*Dicranales*	*Dicranaceae*	*Dicranella*	*Dicranella heteromalla*	1.0	3.6	
**gh_00266**	*Dicranales*	*Dicranaceae*	*Dicranella*	*Dicranella heteromalla*			
**gh_00170**	*Dicranales*	*Dicranaceae*				1.8	15.4
**gh_00170**	*Dicranales*	*Dicranaceae*					
**gh_00245**	*Dipsacales*	*Adoxaceae*	*Viburnum*		1.0		
**gh_00082**	*Equisetales*	*Equisetaceae*	*Equisetum*		2.1	1.8	7.7
**gh_00082**	*Equisetales*	*Equisetaceae*	*Equisetum*				
**gh_00035**	*Ericales*	*Ericaceae*	*Calluna*	*Calluna vulgaris*	2.1	8.9	7.7
**gh_00035**	*Ericales*	*Ericaceae*	*Calluna*	*Calluna vulgaris*			
**gh_00035**	*Ericales*	*Ericaceae*	*Calluna*	*Calluna vulgaris*			
**gh_00110**	*Ericales*	*Ericaceae*	*Empetrum*			1.8	7.7
**gh_00106**	*Ericales*	*Ericaceae*	*Erica*				
**gh_00106**	*Ericales*	*Ericaceae*	*Erica*		2.1	5.4	
**gh_00146**	*Ericales*	*Ericaceae*			3.1		
**gh_00052**	*Ericales*	*Ericaceae*	*Rhododendron*			3.6	
**gh_00090**	*Ericales*	*Ericaceae*	*Vaccinium*		1.0	1.8	
**gh_00047**	*Ericales*	*Ericaceae*	*Vaccinium*	*Vaccinium macrocarpon*		1.8	7.7
**gh_00168**	*Ericales*	*Ericaceae*	*Vaccinium*	*Vaccinium microcarpum*	1.0		7.7
**gh_00142**	*Ericales*	*Ericaceae*	*Vaccinium*	*Vaccinium vitis-idaea*		1.8	
**gh_00050**	*Ericales*	*Primulaceae*	*Lysimachia*		6.3		
**gh_00031**	*Fabales*	*Fabaceae*	*Glycine*			3.6	
**gh_00177**	*Fabales*	*Fabaceae*	*Lathyrus*	*Lathyrus pratensis*	1.0		
**gh_00043**	*Fabales*	*Fabaceae*				1.8	
**gh_00028**	*Fabales*	*Fabaceae*	*Trifolium*		1.0		
**gh_00037**	*Fabales*	*Fabaceae*	*Trifolium*		4.2	3.6	
**gh_00037**	*Fabales*	*Fabaceae*	*Trifolium*				
**gh_00147**	*Fabales*	*Fabaceae*	*Vicia*		2.1		
**gh_00017**	*Fagales*	*Betulaceae*	*Alnus*		20.8	3.6	7.7
**gh_00017**	*Fagales*	*Betulaceae*	*Alnus*				
**gh_00017**	*Fagales*	*Betulaceae*	*Alnus*				
**gh_00008**	*Fagales*	*Betulaceae*			20.8	16.1	7.7
**gh_00008**	*Fagales*	*Betulaceae*					
**gh_00008**	*Fagales*	*Betulaceae*					
**gh_00023**	*Fagales*	*Betulaceae*			1.0		
**gh_00013**	*Fagales*	*Fagaceae*	*Fagus*		16.7	17.9	23.1
**gh_00013**	*Fagales*	*Fagaceae*	*Fagus*				
**gh_00013**	*Fagales*	*Fagaceae*	*Fagus*				
**gh_00020**	*Fagales*	*Fagaceae*	*Fagus*			17.9	23.1
**gh_00020**	*Fagales*	*Fagaceae*	*Fagus*				
**gh_00020**	*Fagales*	*Fagaceae*	*Fagus*		16.7		
**gh_00005**	*Fagales*	*Fagaceae*				7.1	
**gh_00005**	*Fagales*	*Fagaceae*			13.5		
**gh_00007**	*Fagales*	*Fagaceae*	*Quercus*		15.6	1.8	
**gh_00007**	*Fagales*	*Fagaceae*	*Quercus*				
**gh_00129**	*Fagales*	*Juglandaceae*			2.1	1.8	
**gh_00129**	*Fagales*	*Juglandaceae*					

**gh_00034**	*Fagales*	*Myricaceae*				5.4	
**gh_00141**	*Hypnales*	*Brachytheciaceae*			1.0		
**gh_00132**	*Hypnales*				4.2		
**gh_00193**	*Hypnales*				1.0		
**gh_00102**	*Lamiales*	*Lamiaceae*			1.0	1.8	
**gh_00025**	*Lamiales*				1.0		
**gh_00039**	*Lamiales*	*Plantaginaceae*	*Plantago*		5.2	5.4	
**gh_00039**	*Lamiales*	*Plantaginaceae*	*Plantago*				
**gh_00098**	*Malpighiales*	*Hypericaceae*	*Hypericum*			1.8	
**gh_00003**	*Malpighiales*	*Passifloraceae*	*Passiflora*		1.0		
**gh_00009**	*Malpighiales*	*Salicaceae*			1.0	19.6	
**gh_00009**	*Malpighiales*	*Salicaceae*					
**gh_00057**	*Malpighiales*	*Salicaceae*	*Populus*			3.6	7.7
**gh_00057**	*Malpighiales*	*Salicaceae*	*Populus*				
**gh_00053**	*Malvales*	*Malvaceae*	*Tilia*		1.0		
**gh_00267**	*Myrtales*	*Onagraceae*	*Chamerion*	*Chamerion angustifolium*	1.0		
**gh_00296**	*Myrtales*	*Onagraceae*				1.8	
**gh_00048**	*Oxalidales*	*Oxalidaceae*	*Oxalis*		9.4	5.4	
**gh_00048**	*Oxalidales*	*Oxalidaceae*	*Oxalis*				
**gh_00253**	*Pinales*	*Pinaceae*	*Cedrus*			1.8	
**gh_00128**	*Pinales*	*Pinaceae*	*NA*		1.0		
**gh_00012**	*Pinales*	*Pinaceae*	*Picea*		9.4	12.5	15.4
**gh_00012**	*Pinales*	*Pinaceae*	*Picea*				
**gh_00012**	*Pinales*	*Pinaceae*	*Picea*				
**gh_00019**	*Pinales*	*Pinaceae*	*Picea*		5.2	3.6	46.2
**gh_00019**	*Pinales*	*Pinaceae*	*Picea*				
**gh_00019**	*Pinales*	*Pinaceae*	*Picea*				
**gh_00015**	*Pinales*	*Pinaceae*	*Pinus*				
**gh_00015**	*Pinales*	*Pinaceae*	*Pinus*		2.1	7.1	
**gh_00154**	*Poales*	*Cyperaceae*	*Carex*		1.0		
**gh_00228**	*Poales*	*Cyperaceae*	*Carex*		1.0		
**gh_00040**	*Poales*	*Cyperaceae*	*Eriophorum*		1.0	3.6	
**gh_00040**	*Poales*	*Cyperaceae*	*Eriophorum*				
**gh_00075**	*Poales*	*Juncaceae*	*Juncus*		3.1	1.8	
**gh_00075**	*Poales*	*Juncaceae*	*Juncus*				
**gh_00184**	*Poales*	*Juncaceae*	*Luzula*	*Luzula pilosa*	1.0		
**gh_00209**	*Poales*	*Juncaceae*	*Luzula*			1.8	
**gh_00203**	*Poales*	*Poaceae*	*Calamagrostis*		1.0	1.8	
**gh_00054**	*Poales*	*Poaceae*	*Dactylis*	*Dactylis glomerata*	2.1		
**gh_00127**	*Poales*	*Poaceae*	*Holcus*		1.0	1.8	
**gh_00002**	*Poales*	*Poaceae*			18.8	7.1	
**gh_00002**	*Poales*	*Poaceae*					
**gh_00010**	*Poales*	*Poaceae*			10.4	10.7	7.7
**gh_00010**	*Poales*	*Poaceae*					
**gh_00010**	*Poales*	*Poaceae*					
**gh_00016**	*Poales*	*Poaceae*			4.2	7.1	
**gh_00021**	*Poales*	*Poaceae*				3.6	
**gh_00022**	*Poales*	*Poaceae*			9.4	16.1	
**gh_00032**	*Poales*	*Poaceae*			3.1	1.8	

**gh_00032**	*Poales*	*Poaceae*					
**gh_00036**	*Poales*	*Poaceae*			1.0	1.8	
**gh_00049**	*Poales*	*Poaceae*			1.0		
**gh_00081**	*Poales*	*Poaceae*			3.1		
**gh_00116**	*Poales*	*Poaceae*			1.0	3.6	
**gh_00116**	*Poales*	*Poaceae*					
**gh_00171**	*Poales*	*Poaceae*			1.0	3.6	
**gh_00171**	*Poales*	*Poaceae*					
**gh_00280**	*Poales*	*Poaceae*				1.8	
**gh_00070**	*Poales*	*Poaceae*	*Phragmites*	*Phragmites australis*	2.1	1.8	
**gh_00070**	*Poales*	*Poaceae*	*Phragmites*	*Phragmites australis*			
**gh_00067**	*Poales*	*Poaceae*	*Poa*		1.0	5.4	
**gh_00067**	*Poales*	*Poaceae*	*Poa*				
**gh_00108**	*Proteales*	*Platanaceae*	*Platanus*	*Platanus orientalis*		3.6	
**gh_00014**	*Ranunculales*	*Ranunculaceae*	*Ranunculus*				
**gh_00014**	*Ranunculales*	*Ranunculaceae*	*Ranunculus*		4.2	10.7	
**gh_00191**	*Ranunculales*	*Ranunculaceae*	*Ranunculus*		1.0		
**gh_00121**	*Rhizogoniales*	*Orthodontiaceae*	*Orthodontium*	*Orthodontium lineare*		1.8	
**gh_00065**	*Rosales*	*Moraceae*				1.8	15.4
**gh_00065**	*Rosales*	*Moraceae*			3.1		
**gh_00065**	*Rosales*	*Moraceae*					
**gh_00051**	*Rosales*	*Rosaceae*	*Filipendula*	*Filipendula ulmaria*	1.0	3.6	
**gh_00051**	*Rosales*	*Rosaceae*	*Filipendula*	*Filipendula ulmaria*			
**gh_00101**	*Rosales*	*Rosaceae*	*Filipendula*	*Filipendula ulmaria*	1.0		
**gh_00001**	*Rosales*	*Rosaceae*			22.9	21.4	7.7
**gh_00001**	*Rosales*	*Rosaceae*					
**gh_00001**	*Rosales*	*Rosaceae*					
**gh_00018**	*Rosales*	*Rosaceae*				3.6	
**gh_00046**	*Rosales*	*Rosaceae*			2.1	3.6	
**gh_00186**	*Rosales*	*Rosaceae*	*Potentilla*			1.8	
**gh_00011**	*Rosales*	*Rosaceae*	*Prunus*		2.1		7.7
**gh_00011**	*Rosales*	*Rosaceae*	*Prunus*				
**gh_00137**	*Rosales*	*Rosaceae*	*Pyrus*			1.8	
**gh_00084**	*Rosales*	*Rosaceae*	*Rubus*	*Rubus idaeus*	3.1	1.8	
**gh_00084**	*Rosales*	*Rosaceae*	*Rubus*	*Rubus idaeus*			
**gh_00109**	*Rosales*	*Ulmaceae*				5.4	
**gh_00204**	*Sapindales*	*Aceraceae*	*Acer*		1.0		
**gh_00185**	*Saxifragales*	*Haloragaceae*	*Myriophyllum*			1.8	
**gh_00066**	*Solanales*	*Solanaceae*				1.8	
**gh_00288**	*Splachnales*	*Splachnaceae*	*Tetraplodon*	*Tetraplodon pallidus*		1.8	
**gh_00024**	*Zingiberales*	*Musaceae*			3.1	1.8	7.7
**gh_00024**	*Zingiberales*	*Musaceae*					
**mav_00067**	*Agriolimacidae*	*Agriolimacidae*	*Deroceras*	*Deroceras reticulatum*	2.1	1.8	
**mav_00054**	*Arionidae*	*Arionidae*	*Arion*	*Arion circumscriptus*	3.1		
**mav_00182**	*Arionidae*	*Arionidae*	*Arion*	*Arion fuscus*	1.0		
**mav_00036**	*Arionidae*	*Arionidae*	*Arion*	*Arion silvaticus*	5.2		
**mav_00211**	*Arionidae*	*Arionidae*	*Arion*		1.0		
**mav_00031**	*Coleoptera*	*Cantharidae*			1.0		
**mav_00098**	*Coleoptera*	*Chrysomelidae*	*Aspidomorpha*	*Aspidomorpha miliaris*	1.0		
**mav_00007**	*Coleoptera*	*Chrysomelidae*	*Callosobruchus*	*Callosobruchus analis*	6.3	3.6	23.1

**mav_00007**	*Coleoptera*	*Chrysomelidae*	*Callosobruchus*	*Callosobruchus analis*			
**mav_00007**	*Coleoptera*	*Chrysomelidae*	*Callosobruchus*	*Callosobruchus analis*			
**mav_00137**	*Coleoptera*	*Gyrinidae*	*Macrogyrus*	*Macrogyrus oblongus*	2.1		
**mav_00078**	*Coleoptera*	*Melyridae*	*Listrocerus*	*Listrocerus sp. 1186*			7.7
**mav_00008**	*Coleoptera*				15.6		
**mav_00123**	*Coleoptera*					1.8	
**mav_00051**	*Coleoptera*	*Scarabaeidae*					7.7
**mav_00037**	*Coleoptera*	*Staphylinidae*	*Lasinus*	*Lasinus mikado*		3.6	
**mav_00032**	*Coleoptera*	*Staphylinidae*			2.1		
**mav_00026**	*Diptera*	*Chironomidae*	*Paratanytarsus*	*Paratanytarsus grimmii*		1.8	7.7
**mav_00086**	*Diptera*	*Culicidae*	*Culex*	*Culex quinquefasciatus*		3.6	7.7
**mav_00086**	*Diptera*	*Culicidae*	*Culex*	*Culex quinquefasciatus*			
**mav_00092**	*Diptera*	*Culicidae*	*Culex*	*Culex quinquefasciatus*		3.6	15.4
**mav_00190**	*Diptera*	*Culicidae*	*Culex*	*Culex quinquefasciatus*		1.8	
**mav_00239**	*Diptera*	*Culicidae*	*Culex*	*Culex quinquefasciatus*		1.8	
**mav_00029**	*Diptera*	*Lauxaniidae*	*Pseudogriphoneura*	*Pseudogriphoneura sp. ACMJ-2016*	2.1		
**mav_00053**	*Diptera*	*Phoridae*				1.8	
**mav_00060**	*Diptera*	*Sciaridae*			2.1		
**mav_00091**	*Glomerida*	*Glomeridae*	*Glomeris*	*Glomeris marginata*	1.0		
**mav_00096**	*Helocoidea*	*Helicidae*	*Arianta*	*Arianta arbustorum*	1.0		
**mav_00275**	*Helocoidea*	*Helicidae*	*Cepaea*	*Cepaea hortensis*	1.0		
**mav_00115**	*Helocoidea*	*Helicidae*	*Cepaea*		1.0		
**mav_00156**	*Helocoidea*	*Helicidae*	*Cepaea*		2.1		
**mav_00076**	*Hemiptera*	*Cicadellidae*	*Flexamia*		1.0		
**mav_00035**	*Hemiptera*	*Cicadellidae*			1.0		
**mav_00196**	*Hemiptera*	*Cicadellidae*					7.7
**mav_00284**	*Hemiptera*	*Cicadellidae*				1.8	
**mav_00056**	*Hemiptera*					1.8	
**mav_00277**	*Hemiptera*	*Pentatomidae*			1.0		
**mav_00012**	*Hymenoptera*	*Apidae*	*Apis*	*Apis mellifera*	5.2		
**mav_00019**	*Hymenoptera*	*Apidae*	*Bombus*	*NA*	2.1		7.7
**mav_00019**	*Hymenoptera*	*Apidae*	*Bombus*	*NA*			
**mav_00165**	*Hymenoptera*	*Formicidae*	*Camponotus*	*Camponotus herculeanus*	1.0		
**mav_00304**	*Hymenoptera*	*Formicidae*	*Lasius*			1.8	
**mav_00070**	*Hymenoptera*				1.0		
**mav_00129**	*Isopoda*	*Porcellionidae*	*Porcellio*	*Porcellio scaber*		1.8	15.4
**mav_00129**	*Isopoda*	*Porcellionidae*	*Porcellio*	*Porcellio scaber*			
**mav_00090**	*Ixodida*	*Nuttalliellidae*	*Nuttalliella*	*Nuttalliella namaqua*	2.1		
**mav_00099**	*Ixodida*	*Nuttalliellidae*	*Nuttalliella*	*Nuttalliella namaqua*	1.0		
**mav_00081**	*Julida*	*Blaniulidae*	*Proteroiulus*	*Proteroiulus fuscus*			7.7
**mav_00095**	*Lepidoptera*	*Hepialidae*					7.7
**mav_00015**	*Lepidoptera*						
**mav_00015**	*Lepidoptera*				4.2	17.9	
**mav_00122**	*Lepidoptera*					1.8	
**mav_00154**	*Lepidoptera*						7.7
**mav_00220**	*Lepidoptera*					1.8	
**mav_00067**	*Limacoidea*	*Agriolimacidae*	*Deroceras*	*Deroceras reticulatum*			
**mav_00271**	*Nudibranchia*	*Dendrodorididae*	*Doriopsilla*	*Doriopsilla miniata*		1.8	

**mav_00050**	*Nudibranchia*				1.0		7.7
**mav_00111**	*Opiliones*	*Phalangiidae*	*Phalangium*	*Phalangium opilio*	1.0		
**mav_00314**	*Psocoptera*	*Liposcelidae*	*Liposcelis*			1.8	
**mav_00017**	*Thysanoptera*	*Thripidae*	*Scolothrips*	*Scolothrips takahashii*		10.7	
**mav_00072**	*Thysanoptera*	*Thripidae*	*Thrips*	*Thrips flavidulus*			46.2
**v5_00056**	*Anseriformes*	*Anatidae*				1.8	
**v5_00012**	*Anura*	*Bufonidae*	*Bufo*	*Bufo bufo*	6.3	1.8	
**v5_00012**	*Anura*	*Bufonidae*	*Bufo*	*Bufo bufo*			
**v5_00011**	*Anura*	*Ranidae*	*Rana*		12.5		
**v5_00010**	*Anura*	*Ranidae*	*Rana*	*Rana temporaria*	7.3		7.7
**v5_00010**	*Anura*	*Ranidae*	*Rana*	*Rana temporaria*			
**v5_00022**	*Artiodactyla*	*Bovidae*	*Bos*			1.8	
**v5_00028**	*Artiodactyla*	*Cervidae*	*Capreolus*	*Capreolus capreolus*		5.4	
**v5_00004**	*Artiodactyla*	*Cervidae*	*Cervus*	*Cervus elaphus*			
**v5_00004**	*Artiodactyla*	*Cervidae*	*Cervus*	*Cervus elaphus*	12.5	25.0	
**v5_00023**	*Artiodactyla*	*Cervidae*	*Dama*	*Dama dama*		10.7	
**v5_00006**	*Artiodactyla*	*Suidae*	*Sus*	*Sus scrofa*			
**v5_00006**	*Artiodactyla*	*Suidae*	*Sus*	*Sus scrofa*	3.1	39.3	
**v5_00005**	*Carnivora*	*Canidae*	*Nyctereutes*	*Nyctereutes procyonoides*		1.8	7.7
**v5_00008**	*Carnivora*	*Mustelidae*	*Martes*				107.7
**v5_00039**	*Columbiformes*	*Columbidae*	*Columba*				7.7
**v5_00024**	*Galliformes*	*Phasianidae*	*Phasianus*		1.0		
**v5_00064**	*Insectivora*	*Soricidae*	*Sorex*		1.0		
**v5_00020**	*Insectivora*	*Soricidae*	*Sorex*	*Sorex araneus*	1.0		
**v5_00042**	*Insectivora*	*Talpidae*	*Talpa*	*Talpa europaea*	1.0		
**v5_00149**	*Lagomorpha*	*Leporidae*	*Lepus*	*Lepus europaeus*	1.0		
**v5_00159**	*Passeriformes*	*Sturnidae*				1.8	
**v5_00033**	*Passeriformes*	*Turdidae*	*Turdus*	*Turdus philomelos*	1.0		
**v5_00065**	*Pelecaniformes*	*Phalacrocoracidae*	*Phalacrocorax*	*Phalacrocorax carbo*		1.8	
**v5_00007**	*Rodentia*	*Cricetidae*	*Microtus*	*Microtus subterraneus*	13.5	19.6	30.8
**v5_00007**	*Rodentia*	*Cricetidae*	*Microtus*	*Microtus subterraneus*			
**v5_00007**	*Rodentia*	*Cricetidae*	*Microtus*	*Microtus subterraneus*			
**v5_00068**	*Rodentia*	*Cricetidae*	*Microtus*	*Microtus subterraneus*		1.8	
**v5_00026**	*Rodentia*	*Cricetidae*	*Myodes*	*Myodes glareolus*		1.8	
**v5_00015**	*Rodentia*	*Muridae*	*Apodemus*				
**v5_00015**	*Rodentia*	*Muridae*	*Apodemus*		1.0	3.6	
**v5_00034**	*Rodentia*	*Muridae*	*Micromys*	*Micromys minutus*		1.8	7.7
**v5_00074**	*Strigiformes*	*Strigidae*	*Asio*	*Asio otus*		1.8	

Table 5. Taxa identified in badger, fox and marten faecal samples using macroscopic diet analysis. Frequency of Occurrence (FO) is calculated for each taxonomic classification for all predators (badger, N = 96; fox, N = 56; marten, N = 13).

Order	Family	Genus	Species	Badger	Fox	Marten
Poales				16.7	10.7	15.4
Poales	*Poaceae*	*Zea*	*Zea mays*	22.9		
Rosales	*Rosaceae*	*Malus*		6.3	7.1	
Rosales	*Rosaceae*	*Prunus*		2.1	3.6	
Rosales	*Rosaceae*	*Pyrus*		1.0		
Rosales	*Rosaceae*	*Rubus*		2.1		
Rosales	*Rosaceae*	*Sorbus*	*Sorbus aucuparia*			7.7
Coleoptera	*Carabidae*	*Carabua*	*Carabus granulatus*	1.0		
Coleoptera	*Carabidae*			2.1		
Coleoptera	*Chrysomelidae*			1.0		
Coleoptera				84.4	25.0	30.8
Coleoptera	*Scarabaeidae*			2.1		
Coleoptera	*Stayhylinoidea*			2.1		
Diptera	*Muscidae*	*Musa*	*Musa domestica*	1.0		
Diptera				1.0		
Hymenoptera	*Formicidae*			1.0		
Hymenoptera				32.3	7.1	
Isopoda				1.0	1.8	
Squamata	*Lacertidae*			3.1	5.4	
Squamata	*Serpentes*			3.1	3.6	
Squamata	*Viperidae*	*Vipera*	*Vipera berus*	2.1		
Anseriformes					3.6	
Anura				33.3	16.1	
Artiodactyla	*Cervidae*			3.1	51.8	
Artiodactyla	*Suidae*	*Sus*	*Sus scrofa*		1.8	7.7
Carnivora	*Mustelidae*	*Martes*	*Martes foina*		1.8	
Carnivora	*Mustelidae*	*Martes*	*Martes martes*			7.7
Carnivora	*Mustelidae*	*Mustela*	*Mustela lutreola*		1.8	15.4
Carnivora	*Mustelidae*	*Mustela*	*Mustela nivalis*		1.8	
Carnivora	*Mustelidae*				3.6	7.7
Columbiformes						7.7
Erinaceomorpha	*Erinaceidae*	*Erinaceus*			1.8	
Eulipotyphla				1.0		
Eulipotyphla	*Soricidae*			5.2		
Lagomorpha	*Leporidae*	*Lepus*	*Lepus europaeus*	1.0		
Passeriformes				3.1	3.6	7.7
Rodentia	*Cricetidae*	*Arvicola*	*Arvicola amphibius*	6.3		
Rodentia	*Cricetidae*	*Arvicola*	*Arvicola amphibius*		3.6	7.7
Rodentia	*Cricetidae*	*Microtus*	*Microtus agrestis*	2.1	14.3	
Rodentia	*Cricetidae*	*Microtus*	*Microtus agrestis*			30.8
Rodentia	*Cricetidae*	*Microtus*	*Microtus arvalis*			7.7
Rodentia	*Cricetidae*	*Myodes*	*Myodes glareolus*	2.1	1.8	
Rodentia	*Cricetidae*			4.2	17.9	23.1
Rodentia	*Muridae*	*Mus*	*Mus musculus*	1.0		
Rodentia	*Muridae*			1.0	3.6	
Rodentia	*Muridae*	*Rattus*		2.1		
Rodentia				2.1	3.6	

Table 6. Species and orders identified in the diet of red fox, European badger and martens using eDNA metabarcoding, but not observed by field observations. Origin and expected probability of presence in Denmark (based on national species databases).

Order	Species	Distribution	In Denmark?
Acari	Ricinus	Common	Yes
Apiales	Azorella caespitosa	Fakland island (not Europe)	No (family, Apiaceae is in Denmark)
Arionidae	Arion circumscriptus	Common	Yes
Arionidae	Arion fuscus	Europe and Asia	Maybe
Arionidae	Arion silvaticus	Europe	Maybe
Artiodactyla	Dama dama	Common	Yes
Boraginales	Phacelia tanacetifolia	America	Maybe introduced
Carnivora	Mustela lutreola	Not present	No (genus Mustela, and Mustela putorius are found in Denmark)
Caryophyllales	Beta vulgaris	Southeast Europe	Maybe
Coleoptera	Aspidomorpha miliaris	Asia	No (genus, Aspidomorpha is in Denmark)
Coleoptera	Callosobruchus analis	Asia	Maybe introduced
Coleoptera	Lasinus mikado	Japan & Russia	Maybe introduced
Coleoptera	Listrocerus sp. 1186	Information limited	Limited information (Order, Coleoptera is in Denmark)
Coleoptera	Macrogyrus oblongus	Australia	No (family, Gyrinidae, is in Denmark)
Cucurbitales		Agriculturally grown	Yes
Cupressales		Common	Yes
Dicranales	Dicranella heteromalla	American	Yes
Diptera	Culex quinquefasciatus	Africa & south America	No (genus, Culex is in Denmark)
Diptera	Musa domestica	Common	Yes
Diptera	Paratanytarsus grimmii	America	Maybe introduced
Diptera	Pseudogriphoneura sp. ACMJ-2016	Europe	Maybe
Ericales	Vaccinium macrocarpon	America	Introduced
Ericales	Vaccinium microcarpum	Europe	Yes
Erinaceomorpha		Common	Yes
Glomerida	Glomeris marginata	Common	Yes
Helocoidea	Arianta arbustorum	Common	Yes
Helocoidea	Cepaea hortensis	Common	Yes
Hemiptera		Common	Yes
Hymenoptera	Apis mellifera	Common	Yes
Hypnales		Common	Yes
Isopoda	Porcellio scaber	Common	Yes
Ixodida	Nuttalliella namaqua	Africa	Maybe
Julida	Proteroiulus fuscus	Common	Yes
Lepidoptera		Common	Yes
Limacoidea	Deroceras reticulatum	Scandinavia	Yes

Myrtales	Chamerion angustifolium	Common	Yes
Nudibranchia	Doriopsilla miniata	Tropical & subtropical	No (family, Dendrodorididae is in Denmark)
Opiliones	Phalangium opilio	Common	Yes
Poales	Dactylis glomerata	Common	Yes
Poales	Zea mays	Common	Yes
Proteales	Platanus orientalis	Common	Yes
Psocoptera		Common	Yes
Rhizogoniales	Orthodontium lineare	Introduced	Introduced
Rodentia	Microtus arvalis	Common	Yes
Rodentia	Microtus subterraneus	Common	Yes
Rodentia	Myodes glareolus	Common	Yes
Splachnales	Tetraplodon pallidus	American	Maybe introduced
Thysanoptera	Scolothrips takahashii	Common	Yes
Thysanoptera	Thrips flavidulus	Africa, Europe	Maybe introduced
Zingiberales		Tropical	No (could be introduced or left by human*)

* *Zingiberales* covers a series of tropical plants such as the ginger family, banana family etc., so this order could have been present due to human interventions.

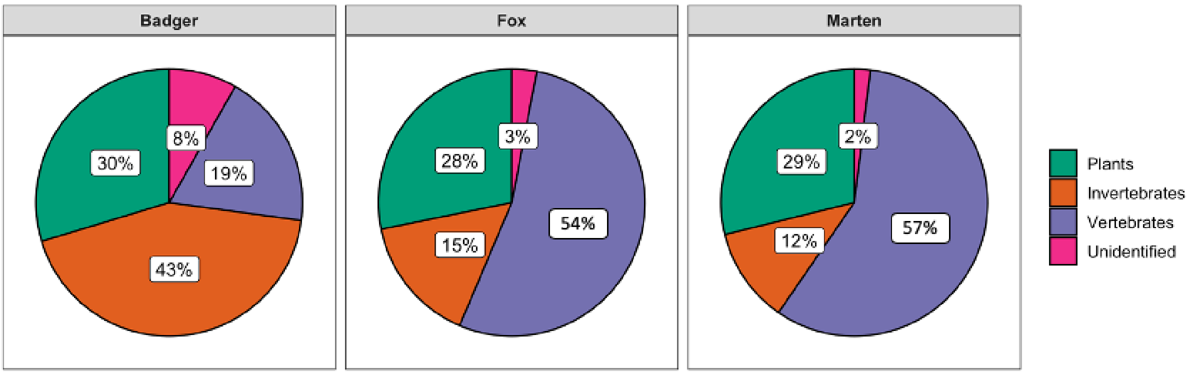

Figure S1. Macroscopic diet composition on major dietary components in badger, fox and marten samples (plant, invertebrate, vertebrate and unidentified).

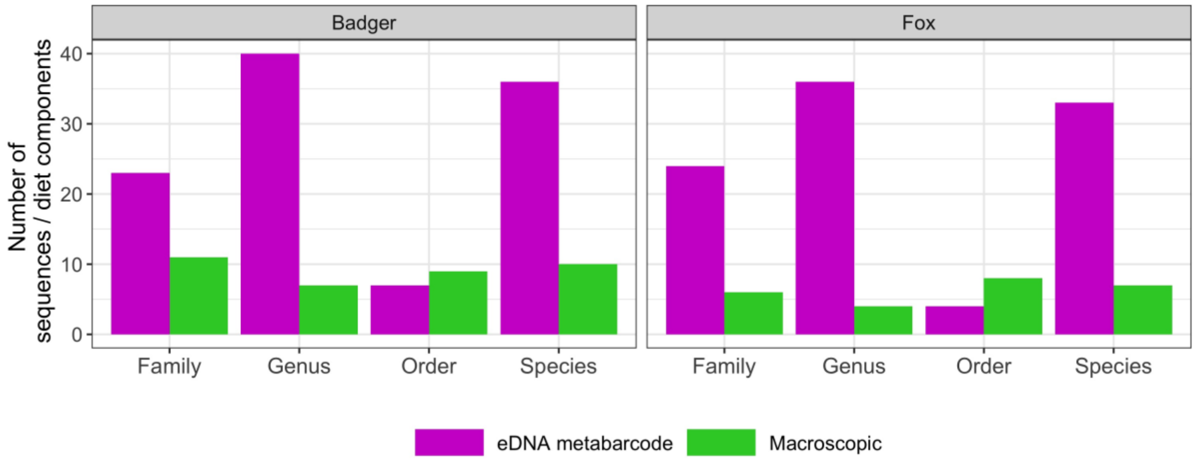

Figure S2. Taxonomic resolution achieved using eDNA metabarcoding and macroscopic diet analysis. Shown are number of different sequences (eDNA metabarcoding) and dietary components (macroscopic diet analysis) identified to either species, genus, family, order, or above order level (*i.e.* class or kingdom) in badger and fox samples.

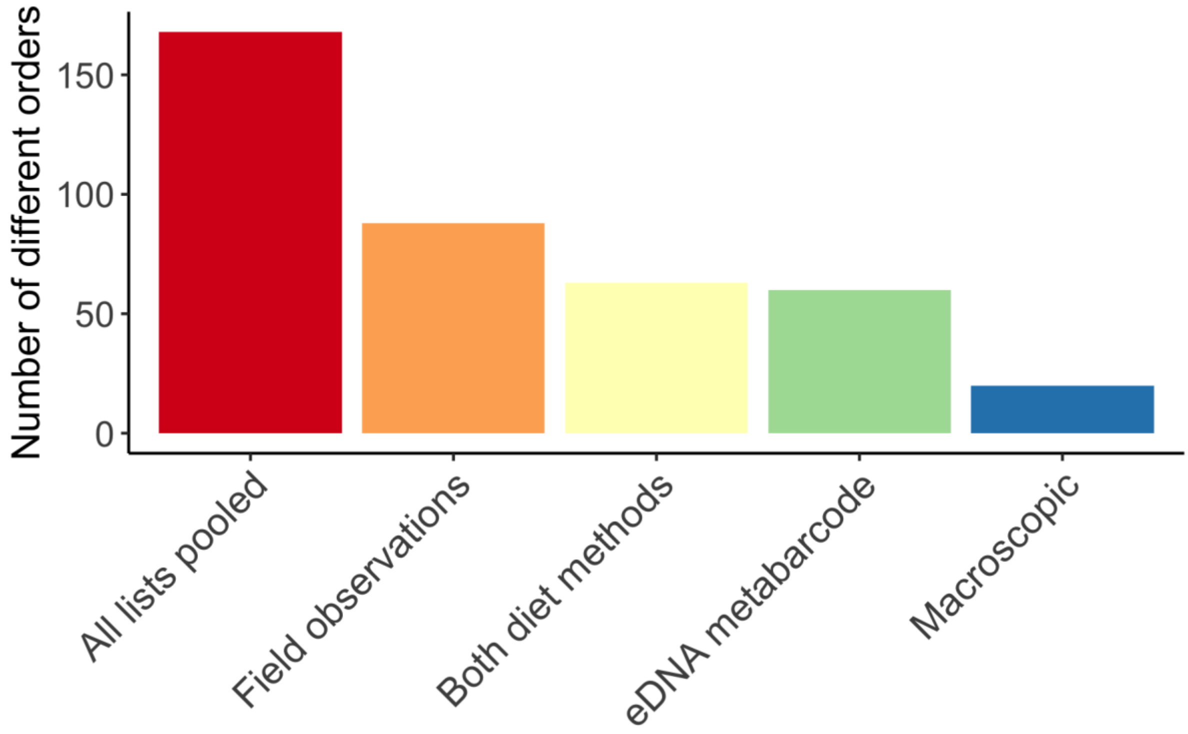

Figure S3. Number of orders detected using the three approaches; all lists (field observations, eDNA metabarcoding and macroscopic diet analysis from badger, fox and marten) pooled, field observations only, both diet assessment methods pooled, and detections using either of the two dietary methods individually.

References

1. Taberlet, P. *et al.* Power and limitations of the chloroplast trnL (UAA) intron for plant DNA barcoding. *Nucleic Acids Res.* **35**, (2007).
2. Taberlet, P., Bonin, A., Zinger, L. & Coissac, E. *Environmental DNA For Biodiversity Research and Monitoring*. (Oxford University Press, 2018).
3. Riaz, T. *et al.* ecoPrimers: inference of new DNA barcode markers from whole genome sequence analysis. *Nucleic Acids Res.* **39**, e145–e145 (2011).
4. De Barba, M. *et al.* DNA metabarcoding multiplexing and validation of data accuracy for diet assessment: Application to omnivorous diet. *Mol. Ecol. Resour.* **14**, 306–323 (2014).
5. Coissac, E. OligoTag: A program for designing sets of tags for next-generation sequencing of multiplexed samples. *Methods Mol. Biol. (Methods Protoc.* **888**, 13–31 (2012).
6. Schnell, I. B. *et al.* Screening mammal biodiversity using DNA from leeches. *Curr. Biol.* **22**, (2012).
7. Boyer, F. *et al.* obitools: a unix-inspired sotfware package for DNA metabarcoding. *Mol. Ecol. Resour.* **16**, 176–182 (2016).
8. R Core Team. *R: A language and environment for statistical computing. R Foundation for Statistical Computing* (2018).
9. Renkonen, O. Statisch-ökologische Untersuchungen über die terrestrische Käferwelt der finnischen Bruchmoore. *Ann. Zool. Soc. Bot. Fenn. Vanamo* **6**, 1–231 (1938).
